# Multimodal spatiotemporal graph convolutional attention network for dynamic risk stratification and intervention strategy generation in rare disease rehabilitation nursing

**DOI:** 10.1038/s41598-026-37095-9

**Published:** 2026-01-30

**Authors:** Siwen Zhao, Min Hu, Shan Fang

**Affiliations:** Shanghai No. 8 Hospital, Xuhui, 200235 Shanghai China

**Keywords:** Rare disease, Rehabilitation nursing, Graph convolutional network, Multimodal fusion, Risk stratification, Attention mechanism, Computational biology and bioinformatics, Diseases, Health care, Mathematics and computing

## Abstract

Rare disease rehabilitation nursing presents unique challenges due to heterogeneous clinical manifestations, limited sample sizes, and complex comorbidity patterns that render traditional risk assessment tools inadequate. This study proposes a novel multimodal spatiotemporal graph convolutional attention network (MSTGCA-Net) for dynamic risk stratification and intervention strategy generation in rare disease rehabilitation. The framework integrates four principal innovations: a heterogeneous patient relationship graph construction scheme encoding clinical similarities, an adaptive multimodal fusion module employing cross-attention mechanisms, a spatiotemporal encoder capturing both inter-patient relationships and longitudinal dependencies, and a knowledge-guided intervention generation component. Experiments conducted on a retrospective cohort of 2,847 patients with 156 rare disease categories demonstrate that MSTGCA-Net achieves superior performance compared to baseline methods, with accuracy of 0.867, F1 score of 0.845, and AUC of 0.923. Expert evaluation of generated intervention strategies yielded favorable assessments across clinical appropriateness, safety, and feasibility dimensions. The attention-based architecture provides interpretable predictions that facilitate clinical adoption. This framework offers promising decision support tools for precision rehabilitation nursing in rare disease populations.

**Supplementary Information:**

The online version contains supplementary material available at 10.1038/s41598-026-37095-9.

## Introduction

Rare diseases, though individually affecting small patient populations, collectively impose substantial burdens on healthcare systems worldwide. The rehabilitation nursing of patients diagnosed with rare conditions presents distinct challenges that differentiate it from conventional chronic disease management^1^. These patients often exhibit highly heterogeneous clinical manifestations, unpredictable disease trajectories, and complex comorbidity patterns that render traditional risk assessment tools inadequate. The scarcity of clinical data further compounds difficulties in developing evidence-based intervention protocols, leaving clinicians to navigate rehabilitation planning with limited guidance.

Risk stratification during rehabilitation constitutes a critical component of patient-centered care, yet current methodologies fall short when applied to rare disease populations. Standard prognostic scoring systems, designed primarily for common conditions with abundant training data, struggle to capture the nuanced risk profiles inherent to rare diseases^2^. Moreover, rehabilitation outcomes depend not only on baseline clinical characteristics but also on dynamic interactions among physiological states, therapeutic responses, and psychosocial factors that evolve throughout the care continuum. This temporal complexity demands analytical frameworks capable of modeling longitudinal dependencies while accommodating irregular observation intervals characteristic of rare disease follow-up.

The emergence of graph neural networks has opened promising avenues for representing complex relational structures in medical data^3^. Unlike conventional deep learning architectures that assume independent and identically distributed samples, graph-based methods explicitly encode relationships among clinical entities—whether patients, symptoms, treatments, or outcomes—within a unified computational framework. Spatiotemporal graph convolutional networks extend this paradigm by incorporating temporal dynamics, enabling simultaneous learning of spatial correlations and sequential patterns^4^. Such capabilities prove particularly valuable for rehabilitation scenarios where treatment effects unfold over time and patient trajectories exhibit meaningful inter-individual similarities.

Multimodal data integration represents another frontier in computational medicine that holds particular relevance for rare disease applications. Rehabilitation nursing generates diverse data streams encompassing structured clinical records, continuous physiological monitoring, functional assessment scales, and qualitative nursing observations^5^. Each modality captures complementary aspects of patient status, yet conventional fusion strategies often fail to exploit cross-modal synergies effectively. Recent advances in attention mechanisms offer sophisticated approaches to modality weighting and feature alignment, allowing models to selectively emphasize the most informative signals for specific predictive tasks^6^. The self-attention paradigm, popularized through transformer architectures, has demonstrated remarkable success across domains ranging from natural language processing to medical image analysis.

Despite these methodological advances, several gaps persist in translating cutting-edge computational techniques to rare disease rehabilitation contexts. First, existing graph-based healthcare models predominantly target acute care settings or common chronic conditions, with limited consideration of the prolonged, variable rehabilitation processes characteristic of rare diseases^7^. Second, multimodal fusion strategies often assume complete data availability across modalities—an assumption rarely satisfied in real-world rare disease cohorts where missing observations and irregular sampling constitute the norm rather than the exception^8^. Third, while risk stratification algorithms continue to mature, the downstream task of generating actionable, personalized intervention recommendations remains relatively underdeveloped. Clinicians require not merely predictions of adverse outcomes but concrete guidance on how rehabilitation plans might be modified to mitigate identified risks.

The rationale for advancing computational approaches in rare disease rehabilitation extends beyond academic interest to pressing clinical imperatives. Healthcare resource constraints demand efficient allocation strategies that prioritize patients at elevated risk while avoiding unnecessary interventions for lower-risk individuals^9^. For rare disease populations specifically, the limited pool of specialized clinical expertise amplifies the value of decision support tools capable of synthesizing disparate information sources into coherent risk assessments. Furthermore, the psychological burden experienced by rare disease patients and their caregivers underscores the importance of transparent, interpretable predictions that facilitate shared decision-making^10^.

This investigation proposes a novel framework integrating multimodal spatiotemporal graph convolutional networks with hierarchical attention mechanisms for dynamic risk stratification and intervention strategy generation in rare disease rehabilitation nursing. The architecture incorporates three principal innovations. First, we design a heterogeneous graph construction scheme that represents patients, clinical events, and rehabilitation milestones as interconnected nodes, with edges encoding semantic and temporal relationships^11^. Second, we develop an adaptive multimodal fusion module employing cross-attention to dynamically weight contributions from different data sources based on their relevance to individualized risk profiles. Third, we introduce an intervention generation component that translates risk predictions into prioritized nursing recommendations through a knowledge-guided decoding mechanism^12^.

The subsequent sections elaborate the technical foundations and empirical validation of this framework. We anticipate that the proposed methodology will contribute meaningfully to precision rehabilitation nursing for rare disease populations, offering clinicians enhanced tools for proactive risk management while respecting the interpretability requirements essential for clinical adoption.

## Theoretical foundations and technical background

### Risk assessment theory in rare disease rehabilitation nursing

Rare diseases, despite their nomenclature suggesting infrequency, collectively affect approximately 300 million individuals globally. Regulatory definitions vary across jurisdictions—the European Union adopts a prevalence threshold of fewer than 5 cases per 10,000 inhabitants, while the United States defines rarity as conditions affecting fewer than 200,000 persons nationally^13^. Over 7,000 distinct rare diseases have been catalogued to date, with genetic etiologies accounting for roughly 80% of documented cases. The remaining conditions arise from infectious agents, autoimmune dysregulation, environmental exposures, or combinations thereof. This etiological diversity translates directly into heterogeneous clinical presentations, variable onset ages, and disparate organ system involvement patterns that complicate standardized approaches to care.

Rehabilitation nursing risk assessment encompasses systematic evaluation of factors that may impede functional recovery or precipitate adverse events during the care process. Traditional frameworks organize assessment along multiple dimensions: physiological stability, functional capacity, psychological adaptation, social support adequacy, and treatment adherence potential^14^. Conventional tools such as the Braden Scale for pressure injury risk or the Morse Fall Scale exemplify domain-specific instruments widely adopted in general rehabilitation settings. However, these standardized instruments presuppose disease trajectories and risk factor distributions derived primarily from common conditions.

The complexity inherent to rare disease rehabilitation stems from several interrelated sources. Patients frequently present with multisystem involvement requiring coordination across numerous specialty services. Symptom fluctuation patterns often defy prediction based on limited population-level evidence. Comorbidity burdens and polypharmacy risks introduce additional layers of clinical uncertainty^15^. Furthermore, the psychosocial dimensions of living with a rare diagnosis—diagnostic odysseys, social isolation, caregiver strain—constitute risk factors inadequately captured by conventional physiological assessments.

Dynamic risk stratification recognizes that patient risk profiles evolve continuously throughout rehabilitation rather than remaining static from admission^16^. This temporal perspective necessitates repeated reassessment and algorithmic updating as new clinical information emerges. The theoretical underpinning draws from survival analysis traditions while incorporating modern concepts of longitudinal phenotyping^17^. Clinical implementation demands computational frameworks capable of integrating streaming data, detecting trajectory inflection points, and recalibrating predictions in near real-time—capabilities that motivate the machine learning approaches elaborated in subsequent sections.

### Graph convolutional networks and Spatiotemporal modeling methods

Graph neural networks represent a paradigm shift in deep learning by extending convolutional operations from regular grid structures to arbitrary graph topologies. Before proceeding, we establish notation conventions used throughout this manuscript. A graph $\:G=\left(V,E\right)$ consists of a node set $\:V$ with $\:\left|V\right|=N$ nodes and an edge set $\:E$ encoding pairwise relationships^18^. The adjacency matrix $\:A\in\:{\mathbb{R}}^{N\times\:N}$ captures connectivity patterns, where $\:{A}_{ij}=1$ indicates an edge between nodes $\:i$ and $\:j$. Node features are organized as a matrix $\:X\in\:{\mathbb{R}}^{N\times\:F}$, with each row representing the $\:F$-dimensional attribute vector of a single node. Temporal indices $\:t\in\:\{\mathrm{1,2},...,T\}$ denote discrete observation time points, with $\:T$ representing the total sequence length for each patient. Superscripts in parentheses denote layer indices in neural network architectures, such that $\:{H}^{\left(l\right)}\in\:{\mathbb{R}}^{N\times\:{d}_{l}}$ represents hidden representations at layer $\:l$ with dimensionality $\:{d}_{l}$. Table 1 provides a comprehensive notation reference.

**Table 1:** Mathematical notation reference.

Symbol	Dimension	Description
$\:N$	Scalar	Number of patient nodes
$\:F$	Scalar	Input feature dimensionality
$\:T$	Scalar	Temporal sequence length
$\:M$	Scalar	Number of data modalities
$\:K$	Scalar	Number of risk categories
$\:d$	Scalar	Hidden representation dimensionality
$\:A$	$\:{\mathbb{R}}^{N\times\:N}$	Adjacency matrix
$\:X$	$\:{\mathbb{R}}^{N\times\:F}$	Node feature matrix
$\:{H}^{\left(l\right)}$	$\:{\mathbb{R}}^{N\times\:{d}_{l}}$	Hidden states at layer $\:l$
$\:{W}^{\left(l\right)}$	$\:{\mathbb{R}}^{{d}_{l-1}\times\:{d}_{l}}$	Weight matrix at layer $\:l$
$\:Q,K,V$	$\:{\mathbb{R}}^{N\times\:{d}_{k}}$	Query, Key, Value matrices
$\:{\alpha\:}_{m}$	Scalar	Modality fusion weight
$\:{\beta\:}_{t,t{\prime\:}}$	Scalar	Temporal attention weight

The foundational graph convolution operation draws from spectral graph theory. The normalized graph Laplacian is defined as:

1 $$\:L={I}_{N}-{D}^{-\frac{1}{2}}A{D}^{-\frac{1}{2}}$$

where $\:D$ denotes the diagonal degree matrix with $\:{D}_{ii}=\sum\:_{j}^{}{A}_{ij}$, and $\:{I}_{N}$ represents the identity matrix^19^. Spectral convolution on graphs operates through eigendecomposition of the Laplacian, expressing $\:L=U\varLambda\:{U}^{T}$ where $\:U$ contains eigenvectors and $\:\varLambda\:$ holds corresponding eigenvalues. A spectral graph filter can then be formulated as:

2 $$\:{g}_{\theta\:}\mathrm{*}x=U{g}_{\theta\:}\left(\varLambda\:\right){U}^{T}x$$

This formulation, while theoretically elegant, incurs prohibitive computational costs for large graphs. The seminal work on graph convolutional networks addressed this limitation through Chebyshev polynomial approximation^20^. The $\:K$-order approximation yields:

3 $$\:{g}_{{\theta\:}^{{\prime\:}}}\mathrm{*}x\approx\:\sum\:_{k=0}^{K}{\theta\:}_{k}^{{\prime\:}}{T}_{k}\left(\stackrel{\sim}{L}\right)x$$

where $\:{T}_{k}$ denotes Chebyshev polynomials and $\:\stackrel{\sim}{L}=\frac{2}{{\lambda\:}_{max}}L-{I}_{N}$ represents the scaled Laplacian. Further simplification with $\:K=1$ produces the widely adopted propagation rule:

4 $$\:{H}^{(l+1)}=\sigma\:\left({\stackrel{\sim}{D}}^{-\frac{1}{2}}\stackrel{\sim}{A}{\stackrel{\sim}{D}}^{-\frac{1}{2}}{H}^{\left(l\right)}{W}^{\left(l\right)}\right)$$

Here $\:\stackrel{\sim}{A}=A+{I}_{N}$ incorporates self-loops, $\:\stackrel{\sim}{D}$ is the corresponding degree matrix, $\:{W}^{\left(l\right)}$ contains learnable parameters, and $\:\sigma\:$ applies a nonlinear activation function.

Spatiotemporal graph convolutional networks extend this framework to dynamic settings where both graph structure and node features evolve over time. Given a sequence of graph signals $\:\mathcal{X}=\{{X}_{1},{X}_{2},...,{X}_{T}\}$ across $\:T$ time steps, the spatiotemporal convolution jointly captures spatial dependencies and temporal dynamics^21^. A typical formulation decomposes the operation as:

5 $$\:{Z}_{t}={f}_{spatial}({X}_{t},A)$$

6 $$\:Y={f}_{temporal}({Z}_{1},{Z}_{2},...,{Z}_{T})$$

The temporal component commonly employs gated recurrent units or temporal convolutional layers. One effective temporal gating mechanism follows:

7 $$\:{h}_{t}=(1-{u}_{t})\odot\:{h}_{t-1}+{u}_{t}\odot\:{\stackrel{\sim}{h}}_{t}$$

where $\:{u}_{t}$ represents the update gate and $\:\odot\:$ denotes element-wise multiplication. The combined spatiotemporal representation emerges as:

8 $$\:\mathcal{H}=\varphi\:({W}_{s}{\mathrm{*}}_{G}X+{W}_{t}{\mathrm{*}}_{T}X+b)$$

where $\:{\mathrm{*}}_{G}$ and $\:{\mathrm{*}}_{T}$ represent graph and temporal convolutions respectively^22^.

Healthcare applications have witnessed growing adoption of these architectures. Clinical event prediction, patient similarity networks, and disease progression modeling all benefit from explicit relational reasoning^23^. The capacity to model irregular temporal patterns and heterogeneous patient relationships positions spatiotemporal graph networks as particularly promising tools for rehabilitation outcome prediction.

### Multimodal fusion and attention mechanisms

Clinical environments generate remarkably diverse data streams that collectively paint a comprehensive portrait of patient status. Structured electronic health records capture demographic variables, diagnostic codes, and laboratory values, while unstructured clinical notes document nuanced observations that resist standardized encoding^24^. Physiological monitoring contributes continuous waveform data—heart rhythms, respiratory patterns, movement trajectories—that reveal dynamic fluctuations invisible in discrete measurements. Medical imaging adds spatial anatomical information, and increasingly, genomic profiles inform personalized treatment considerations. Each modality possesses distinct statistical properties, dimensionality characteristics, and sampling frequencies that must be reconciled during computational integration.

Fusion strategies occupy a spectrum from early to late integration approaches. Early fusion concatenates raw or minimally processed features from all modalities into a unified representation prior to model training. Given modality-specific feature vectors $\:{x}_{1},{x}_{2},...,{x}_{M}$ for $\:M$ modalities, early fusion produces:

9 $$\:{z}_{early}=f\left(\right[{x}_{1}\oplus\:{x}_{2}\oplus\:\cdots\:\oplus\:{x}_{M}\left]\right)$$

where $\:\oplus\:$ denotes concatenation and $\:f$ represents a shared learning function^25^. Late fusion, conversely, trains modality-specific models independently before combining predictions at the decision level. Hybrid approaches seek intermediate ground by allowing cross-modal interaction at selected processing stages while preserving modality-specific pathways.

Attention mechanisms have fundamentally transformed how neural architectures weight information contributions. The scaled dot-product attention computes relevance scores between query and key vectors:

10 $$\:\mathrm{A}\mathrm{t}\mathrm{t}\mathrm{e}\mathrm{n}\mathrm{t}\mathrm{i}\mathrm{o}\mathrm{n}(Q,K,V)=\mathrm{s}\mathrm{o}\mathrm{f}\mathrm{t}\mathrm{m}\mathrm{a}\mathrm{x}\left(\frac{Q{K}^{T}}{\sqrt[]{{d}_{k}}}\right)V$$

Here $\:Q$, $\:K$, and $\:V$ denote query, key, and value matrices respectively, while $\:{d}_{k}$ represents the key dimensionality^26^. The scaling factor $\:\sqrt[]{{d}_{k}}$ prevents gradient instability when dot products grow large. Self-attention emerges when queries, keys, and values all derive from the same input sequence, enabling each position to attend to all others:

11 $$\:Q=X{W}^{Q},K=X{W}^{K},V=X{W}^{V}$$

Multi-head attention extends this formulation by projecting inputs into multiple parallel subspaces:

12 $$\:\mathrm{M}\mathrm{u}\mathrm{l}\mathrm{t}\mathrm{i}\mathrm{H}\mathrm{e}\mathrm{a}\mathrm{d}(Q,K,V)=\mathrm{C}\mathrm{o}\mathrm{n}\mathrm{c}\mathrm{a}\mathrm{t}(hea{d}_{1},...,hea{d}_{h}){W}^{O}$$

Each head computes independent attention with its own parameter matrices:

13 $$\:head_{i} = {\mathrm{Attention}}(QW_{i}^{Q} ,KW_{i}^{K} ,VW_{i}^{V} )$$

Attention weights offer one useful lens for inspecting model behavior in clinical applications. Unlike fully opaque deep learning architectures, attention scores provide some indication of which input elements the model weighted during prediction^27^. Clinicians may examine these weights to explore whether model focus patterns appear consistent with established medical knowledge—a capability increasingly relevant given regulatory interest in algorithmic transparency^28^.

However, recent research has raised important caveats regarding attention-based interpretability that warrant acknowledgment. Several studies have demonstrated that attention weights do not necessarily correspond to causal importance or true decision influence, and that alternative attention distributions can sometimes yield identical predictions^48^. Attention visualizations should therefore be understood as one exploratory tool among several, rather than definitive explanations of model reasoning. This perspective informs our subsequent evaluation strategy, where we complement attention analysis with expert clinical review to assess whether highlighted patterns appear genuinely informative or merely visually plausible. Such limitations underscore the need for continued development of rigorous interpretability methods in healthcare AI.

## Construction of multimodal Spatiotemporal graph convolutional attention network model

### Multimodal data representation and patient relationship graph construction

Rare disease rehabilitation nursing demands comprehensive data acquisition spanning multiple clinical domains to adequately characterize patient complexity. Our framework integrates four principal data modalities that collectively capture physiological dynamics, anatomical status, clinical narratives, and functional outcomes^29^. Physiological indicators encompass vital signs recorded at regular intervals—heart rate variability, blood pressure fluctuations, oxygen saturation trends, and respiratory patterns—alongside laboratory biomarkers reflecting metabolic and inflammatory states. Medical imaging contributes structural and functional information through modalities such as magnetic resonance imaging, computed tomography, and ultrasound examinations pertinent to specific rare disease manifestations. Electronic health record texts document clinical reasoning, symptom progression, medication adjustments, and nursing observations in unstructured narrative form. Standardized rehabilitation assessment scales quantify functional capacity, pain levels, quality of life, and activity limitations through validated instruments.

Table 2 summarizes the characteristics of each data modality incorporated within our multimodal fusion framework, detailing dimensionality, temporal resolution, and preprocessing requirements that inform subsequent feature engineering decisions.

**Table 2:** Multimodal data feature description for rare disease rehabilitation.

Data modality	Feature dimension	Sampling frequency	Data format	Preprocessing method
Vital signs	12	Continuous/hourly	Numeric	Z-score normalization
Laboratory results	48	Irregular	Numeric	Min-max scaling
Medical imaging	512	Per examination	Tensor	CNN feature extraction
Clinical notes	768	Per encounter	Text	BERT embedding
Rehabilitation scales	24	Weekly	Ordinal	Ordinal encoding
Medication records	156	Daily	Categorical	One-hot encoding
Demographic data	18	Static	Mixed	Hybrid encoding
Comorbidity profiles	64	Admission	Binary	Binary vectorization

The multimodal patient representation emerges through modality-specific encoding followed by unified embedding projection. For patient $\:i$, we denote modality-specific feature vectors as $\:{x}_{i}^{\left(m\right)}$ where $\:m\in\:\{\mathrm{1,2},...,M\}$ indexes data sources. The consolidated patient embedding applies learned transformations:

14 $$\:h_{i} = \sum\limits_{{m = 1}}^{M} {\alpha _{m} \cdot g_{m} \left( {x_{i}^{{\left( m \right)}} } \right)}$$

where $\:g_{m} ( \cdot )$ represents modality-specific encoding networks and $\:{\alpha\:}_{m}$ denotes learnable fusion weights^30^. This weighted aggregation permits adaptive emphasis on modalities most informative for individual patient contexts.

Patient relationship graph construction proceeds by quantifying pairwise similarities across the cohort. We compute composite similarity scores that integrate clinical phenotype overlap, treatment trajectory concordance, and demographic proximity. The clinical similarity function $\:\varphi\:$ combines multiple feature domains through a weighted scheme:

15 $$\:\varphi\:\left(i,j\right)=\sum\:_{d=1}^{D}{\omega\:}_{d}\cdot\:{\mathrm{sim}}_{d}\left({x}_{i}^{\left(d\right)},{x}_{j}^{\left(d\right)}\right)$$

where $\:D$ denotes the number of clinical domains (diagnosis codes, laboratory patterns, medication profiles, and functional scores), $\:{\omega\:}_{d}$ represents domain-specific weights learned during training, and $\:{\mathrm{sim}}_{d}\left(\cdot\:,\cdot\:\right)$ computes cosine similarity within each domain. This formulation allows the model to adaptively emphasize domains most relevant for establishing clinically meaningful patient relationships.

The edge weight between patients $\:i$ and $\:j$ follows:

16 $$\:{w}_{ij}=\mathrm{e}\mathrm{x}\mathrm{p}\left(-\frac{\parallel\:{h}_{i}-{h}_{j}{\parallel\:}^{2}}{2{\sigma\:}^{2}}\right)\cdot\:1\left[\varphi\:\left(i,j\right)>\tau\:\right]$$

Here $\:\sigma\:\in\:{\mathbb{R}}^{+}$ controls the kernel bandwidth, $\:\varphi\:\left(i,j\right)$ measures composite clinical similarity as defined above, and $\:\tau\:\in\:\left[\mathrm{0,1}\right]$ establishes a sparsification threshold preventing spurious connections^31^. The indicator function $\:1\left[\cdot\:\right]$ ensures only meaningfully related patients share edges.

We determined optimal values for $\:\sigma\:$ and $\:\tau\:$ through systematic grid search on the validation set. Table 3 presents the sensitivity analysis examining how model performance varies with different graph construction parameters. The kernel bandwidth $\:\sigma\:$ was searched over $\:\left\{\mathrm{0.5,1.0,2.0,5.0}\right\}$, while the sparsification threshold $\:\tau\:$ ranged from $\:0.1$ to $\:0.5$ in increments of $\:0.1$.

**Table 3:** Sensitivity analysis of graph construction hyperparameters.

Parameter	Value	Graph density	F1 score	AUC	Stability
σ = 0.5	τ = 0.3	0.12	0.821	0.901	0.89
σ = 1.0	τ = 0.3	0.18	0.838	0.912	0.92
σ = 2.0	τ = 0.3	0.24	0.845	0.923	0.94
σ = 5.0	τ = 0.3	0.31	0.829	0.908	0.87
σ = 2.0	τ = 0.1	0.42	0.812	0.895	0.81
σ = 2.0	τ = 0.2	0.32	0.831	0.911	0.88
σ = 2.0	τ = 0.4	0.15	0.836	0.915	0.91
σ = 2.0	τ = 0.5	0.08	0.798	0.882	0.85

The results indicate that moderate graph density (approximately 0.20–0.25) yields optimal performance, with $\:\sigma\:=2.0$ and $\:\tau\:=0.3$ producing the best balance between connectivity and sparsity. Excessively dense graphs ($\:\tau\:=0.1$) introduce noisy connections that degrade prediction quality, while overly sparse graphs ($\:\tau\:=0.5$) limit beneficial information propagation across similar patients. These findings demonstrate that performance gains stem from the architectural design rather than fortuitous parameter selection, as the model maintains robust performance across a reasonable parameter range.

**Fig. 1:**
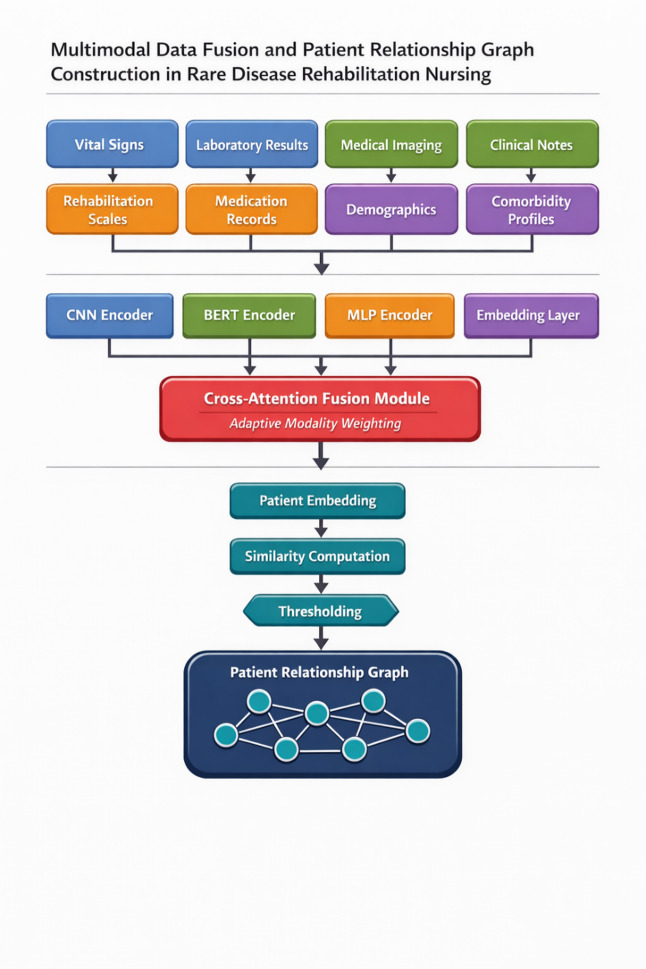
Flowchart of multimodal data fusion and patient relationship graph construction. Input data streams from four modalities (vital signs: $\:{\mathbb{R}}^{12}$, laboratory results: $\:{\mathbb{R}}^{48}$, imaging features: $\:{\mathbb{R}}^{512}$, clinical text embeddings: $\:{\mathbb{R}}^{768}$) flow through modality-specific encoders (CNN, BERT, MLP) producing standardized representations ($\:{\mathbb{R}}^{256}$ each). Cross-attention fusion (center) combines modality representations with learned weights $\:{\alpha\:}_{m}$ into unified patient embeddings $\:{h}_{i}\in\:{\mathbb{R}}^{256}$. The similarity computation module (right) calculates pairwise patient affinities using Eq. (15), with thresholding ($\:\tau\:=0.3$) and kernel transformation ($\:\sigma\:=2.0$) producing the sparse adjacency matrix $\:A\in\:{\mathbb{R}}^{N\times\:N}$. Arrows indicate data flow direction; dashed lines represent optional pathways activated during training only.

Dynamic graph updating accommodates temporal evolution in patient relationships as rehabilitation progresses. At each time step $\:t$, the adjacency matrix recalculates based on current feature representations:

17 $$\:{A}_{t}={f}_{graph}({H}_{t},{\varTheta\:}_{adj})$$

where $\:{H}_{t}$ aggregates patient embeddings at time $\:t$ and $\:{\varTheta\:}_{adj}$ contains graph construction parameters^32^. As illustrated in Fig. 1, the complete pipeline transforms heterogeneous clinical inputs through modality-specific encoders, fuses representations via attention-weighted combination, and constructs the patient relationship graph through similarity computation and thresholding operations.

### Design of spatiotemporal graph convolutional attention encoder

**Fig. 2:**
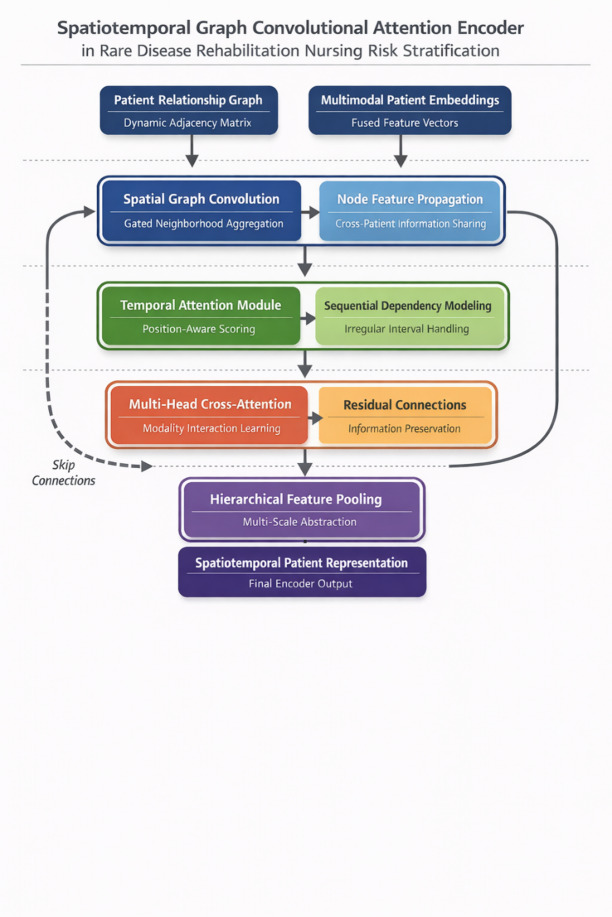
Architecture of the spatiotemporal graph convolutional attention encoder. The encoder comprises three hierarchical levels processing patient representations across spatial (graph) and temporal dimensions. Level 1 (bottom) applies 3-layer graph convolution on patient similarity graphs, transforming node features from $\:{\mathbb{R}}^{256}$ to $\:{\mathbb{R}}^{256}$ via gated aggregation (Eq. 17). Level 2 (middle) implements 8-head temporal attention over $\:T=12$ time steps, with relative position encoding $\:\varphi\:\left(t-t{\prime\:}\right)$. Level 3 (top) performs cross-modal attention between modality-specific pathways. Skip connections (vertical arrows) bridge resolution levels. Output dimensionality: $\:{H}^{global}\in\:{\mathbb{R}}^{N\times\:256}$. Tensor dimensions annotated at each module interface.

The encoder architecture must simultaneously address two intertwined challenges: extracting meaningful patterns from patient relationship structures and modeling temporal dependencies inherent to rehabilitation trajectories. Our design philosophy prioritizes modularity—permitting independent optimization of spatial and temporal components—while ensuring seamless information flow between processing stages. The resulting framework, depicted in Fig. 2, arranges specialized computational blocks in a hierarchical configuration that progressively abstracts from local interactions to global patient representations.

The spatial graph convolution module operates on the patient relationship graph constructed in Sect. 3.1, propagating information across clinically similar patients to enrich individual representations. At each layer, node features aggregate neighborhood information weighted by edge strengths. We adopt a gated aggregation scheme that selectively filters incoming messages:

18 $$\:{z}_{i}^{\left(l\right)}=\sigma\:\left(\sum\:_{j\in\:\mathcal{N}\left(i\right)}^{}\frac{{w}_{ij}}{\sqrt[]{{d}_{i}{d}_{j}}}{W}^{\left(l\right)}{h}_{j}^{(l-1)}+{b}^{\left(l\right)}\right)\odot\:\mathrm{t}\mathrm{a}\mathrm{n}\mathrm{h}\left(\sum\:_{j\in\:\mathcal{N}\left(i\right)}^{}\frac{{w}_{ij}}{\sqrt[]{{d}_{i}{d}_{j}}}{U}^{\left(l\right)}{h}_{j}^{(l-1)}\right)$$

Here $\:\mathcal{N}\left(i\right)$ denotes the neighborhood of patient $\:i$, $\:{d}_{i}$ represents node degree, and the gating mechanism controlled by $\:\sigma\: ( \cdot )$ modulates information flow^33^. This formulation proves particularly valuable for rare disease cohorts where patient subgroups may exhibit divergent response patterns requiring selective knowledge transfer.

Temporal dynamics demand equally careful treatment. Rehabilitation unfolds across extended time horizons with clinically meaningful events—therapy sessions, medication changes, functional assessments—occurring at irregular intervals. Our temporal attention module adapts to these irregularities through position-aware attention scoring. Given a sequence of patient states $\:\{{h}_{1},{h}_{2},...,{h}_{T}\}$, temporal attention weights compute as:

19 $$\:{\beta\:}_{t,{t}^{{\prime\:}}}=\frac{\mathrm{e}\mathrm{x}\mathrm{p}\left(\left({W}_{q}{h}_{t}{)}^{T}\right({W}_{k}{h}_{{t}^{{\prime\:}}})/\sqrt[]{d}+\varphi\:(t-{t}^{{\prime\:}})\right)}{\sum\:_{s=1}^{T}\mathrm{e}\mathrm{x}\mathrm{p}\left(\left({W}_{q}{h}_{t}{)}^{T}\right({W}_{k}{h}_{s})/\sqrt[]{d}+\varphi\:(t-s)\right)}$$

The function $\:\varphi\: ( \cdot )$ encodes relative temporal distances, enabling the model to distinguish recent observations from distant historical records^34^. Temporally attended representations then emerge through weighted aggregation:

20 $$\:{\stackrel{\sim}{h}}_{t}=\sum\:_{{t}^{{\prime\:}}=1}^{T}{\beta\:}_{t,{t}^{{\prime\:}}}\left({W}_{v}{h}_{{t}^{{\prime\:}}}\right)$$

Cross-modal interactions receive explicit modeling through multi-head cross-attention mechanisms. Different data modalities carry complementary information—physiological signals capture acute fluctuations while imaging reveals structural changes—and their optimal combination varies across patients and time points. We formulate cross-modal attention with modality $\:m$ attending to modality $\:n$ as:

21 $$\:{C}^{(m,n)}=\mathrm{s}\mathrm{o}\mathrm{f}\mathrm{t}\mathrm{m}\mathrm{a}\mathrm{x}\left(\frac{{Q}^{\left(m\right)}{{K}^{\left(n\right)}}^{T}}{\sqrt[]{{d}_{k}}}\right){V}^{\left(n\right)}$$

Multiple attention heads operate in parallel, each potentially discovering distinct cross-modal relationships^35^. The outputs concatenate and project to the hidden dimension, with residual connections preserving modality-specific information that might otherwise be obscured during fusion.

The hierarchical encoder structure organizes these components across multiple resolution levels. Lower layers focus on local temporal windows and immediate graph neighborhoods, capturing fine-grained clinical dynamics. Higher layers progressively expand receptive fields—both spatially across the patient graph and temporally across the rehabilitation timeline—distilling global patterns. Skip connections bridge resolution levels:

22 $$\:{H}^{global}=\mathrm{P}\mathrm{o}\mathrm{o}\mathrm{l}\left({H}^{local}\right)+\mathrm{T}\mathrm{r}\mathrm{a}\mathrm{n}\mathrm{s}\mathrm{f}\mathrm{o}\mathrm{r}\mathrm{m}\left({H}^{\left(L\right)}\right)$$

This multi-scale design proves essential for rare disease applications where clinically significant signals may manifest at vastly different temporal scales—from hour-to-hour physiological variations to month-long functional recovery trajectories^36^. The pooling operation abstracts local representations while the transformation adapts the final layer output, together yielding comprehensive patient embeddings suitable for downstream risk stratification tasks.

### Dynamic risk stratification and intervention strategy generation module

Risk stratification transforms the learned patient representations into clinically actionable categories that guide care intensity decisions. The classifier receives spatiotemporal embeddings from the encoder and projects them through successive nonlinear transformations before producing probabilistic risk assignments. We adopt a multi-layer architecture with batch normalization and dropout regularization to prevent overfitting—a concern particularly acute given the limited sample sizes typical of rare disease cohorts^37^. The final softmax layer outputs a probability distribution across $\:K$ predefined risk strata:

23 $$\:P(y=k|{h}_{i}^{\left(T\right)})=\frac{\mathrm{e}\mathrm{x}\mathrm{p}({W}_{k}^{T}{h}_{i}^{\left(T\right)}+{b}_{k})}{\sum\:_{j=1}^{K}\mathrm{e}\mathrm{x}\mathrm{p}({W}_{j}^{T}{h}_{i}^{\left(T\right)}+{b}_{j})}$$

Beyond point predictions, reliable clinical deployment demands honest uncertainty quantification. We implement Monte Carlo dropout at inference time, generating multiple stochastic forward passes to estimate predictive variance. High variance signals cases where model confidence remains low—perhaps due to atypical presentations or data quality issues—alerting clinicians to exercise heightened judgment.

Intervention strategy generation extends beyond passive risk prediction toward active recommendation of therapeutic adjustments. We conceptualize this task as conditional sequence generation, where the model produces structured intervention plans contingent on current patient state and predicted risk trajectory. The generator receives the patient embedding concatenated with the risk distribution and autoregressively decodes intervention components:

24 $$\:p\left(I\right|h,y)=\prod\:_{t=1}^{L}p\left({i}_{t}\right|{i}_{<t},h,y)$$

Each intervention token $\:{i}_{t}$ represents a discrete clinical action—medication modification, therapy intensity adjustment, monitoring frequency change, or specialist consultation recommendation^38^. The vocabulary of permissible actions derives from clinical expert consultation and guideline review, ensuring generated strategies remain within established practice boundaries.

Clinical feasibility constraints impose additional structure on the generation process. Certain intervention combinations prove contraindicated or logistically impractical; the model must respect these restrictions. We encode constraints as a compatibility matrix $\:C$ where $\:{C}_{ij}=0$ indicates interventions $\:i$ and $\:j$ cannot co-occur. During decoding, masked softmax zeroes out probabilities for constraint-violating actions:

25 $$\:p^{\prime}(i_{t} |i_{{ < t}} ,h,y) = \frac{{p(i_{t} |i_{{ < t}} ,h,y) \cdot 1[\forall s < t:C_{{i_{s} ,i_{t} }} = 1]}}{{\sum\nolimits_{j} {p(j|i_{{ < t}} ,h,y) \cdot 1[\forall s < t:C_{{i_{s} ,j}} = 1]} }}$$

End-to-end training optimizes risk stratification and intervention generation jointly through a composite loss function. The multi-task objective balances classification accuracy against generation quality:

26 $$\:{\mathcal{L}}_{total}={\lambda\:}_{1}{\mathcal{L}}_{CE}(y\hat ,{y})+{\lambda\:}_{2}{\mathcal{L}}_{NLL}(I\hat ,{I})+{\lambda\:}_{3}{\mathcal{L}}_{reg}$$

Here $\:{\mathcal{L}}_{CE}$ denotes cross-entropy loss for risk classification, $\:{\mathcal{L}}_{NLL}$ represents negative log-likelihood for intervention sequence generation, and $\:{\mathcal{L}}_{reg}$ encompasses regularization terms^39^. The hyperparameters $\:{\lambda\:}_{1}$, $\:{\lambda\:}_{2}$, and $\:{\lambda\:}_{3}$ control relative task weighting, with values determined through validation set optimization. Gradient accumulation across both task-specific losses enables shared encoder parameters to learn representations beneficial for both objectives, while task-specific decoder components specialize for their respective outputs.

## Experimental design and results analysis

### Dataset and experimental settings

Our experimental validation draws upon a retrospective cohort assembled from three tertiary medical centers specializing in rare disease management between January 2018 and December 2023. The dataset encompasses 2,847 patients diagnosed with 156 distinct rare conditions spanning metabolic disorders, neuromuscular diseases, immunodeficiency syndromes, and connective tissue abnormalities. Each patient contributed longitudinal records covering rehabilitation episodes ranging from 4 weeks to 18 months, yielding 47,362 individual observation time points. Institutional review boards at all participating centers granted ethical approval prior to data extraction, with patient consent obtained according to local regulations governing retrospective clinical research^40^.

Table 4 summarizes the principal statistical characteristics of the assembled dataset, revealing moderate class imbalance across risk strata and substantial variability in observation density. Risk categories were operationally defined based on composite clinical endpoints: High Risk denoted patients experiencing adverse events (unplanned hospitalization, functional decline exceeding 20%, or mortality) within 90 days; Moderate-High Risk indicated elevated biomarker trajectories or functional decline between 10 and 20%; Moderate-Low Risk represented stable patients with minor fluctuations; and Low Risk characterized patients demonstrating consistent improvement. These definitions were established through consensus among clinical collaborators prior to model development, with labels assigned retrospectively based on documented outcomes.

Disease categories were distributed across data splits using stratified sampling to ensure proportional representation, though some rare subtypes appeared in fewer than all three partitions due to extremely limited sample sizes. Specifically, 17 disease categories with fewer than 10 total patients appeared in training only, preventing direct test-set evaluation for these conditions.

**Table 4:** Statistical characteristics of the rare disease rehabilitation dataset.

Characteristic	Training set	Validation set	Test set
Patient count	1993	427	427
Total observations	33,154	7104	7104
Mean follow-up (weeks)	24.6 ± 12.3	23.8 ± 11.9	25.1 ± 12.7
High risk proportion	18.3%	17.9%	18.7%
Moderate-high risk	24.1%	23.6%	24.3%
Moderate-low risk	31.2%	32.1%	30.8%
Low risk	26.4%	26.4%	26.2%
Missing data rate	12.4%	11.8%	12.1%
Disease categories	156	142	139
Overlapping categories	–	138	134

Data preprocessing addressed quality issues inevitable in real-world clinical records. Missing values received imputation through multiple strategies tailored to data characteristics and clinical considerations. For vital signs exhibiting temporal continuity, we employed last observation carried forward given the physiological rationale that recent measurements typically approximate current status. Laboratory values, which display greater variability and disease-specific patterns, received median imputation computed within disease category groups rather than globally, partially accounting for condition-specific reference ranges^41^. Text fields used masked token insertion to preserve sequence structure while signaling missing information to downstream encoders.

We recognize that standard imputation approaches may not optimally suit all 156 rare disease categories, as different conditions exhibit distinct missing data mechanisms and value distributions. To assess imputation sensitivity, we compared disease-stratified median imputation against global median and multiple imputation by chained equations (MICE) on a validation subset. Disease-stratified imputation improved F1 score by 2.1% over global median imputation for metabolic disorders but showed negligible difference for neuromuscular conditions, suggesting heterogeneous imputation effects across disease families. Table 4 reports the overall missing data rates; Supplementary File 1 provides disease-category-specific missing patterns and imputation validation results.

Outlier detection applied isolation forest algorithms to continuous variables, with flagged values reviewed against clinical plausibility before replacement or retention. Feature standardization normalized continuous inputs to zero mean and unit variance within the training partition, with transformation parameters subsequently applied to validation and test sets to prevent information leakage.

**Fig. 3:**
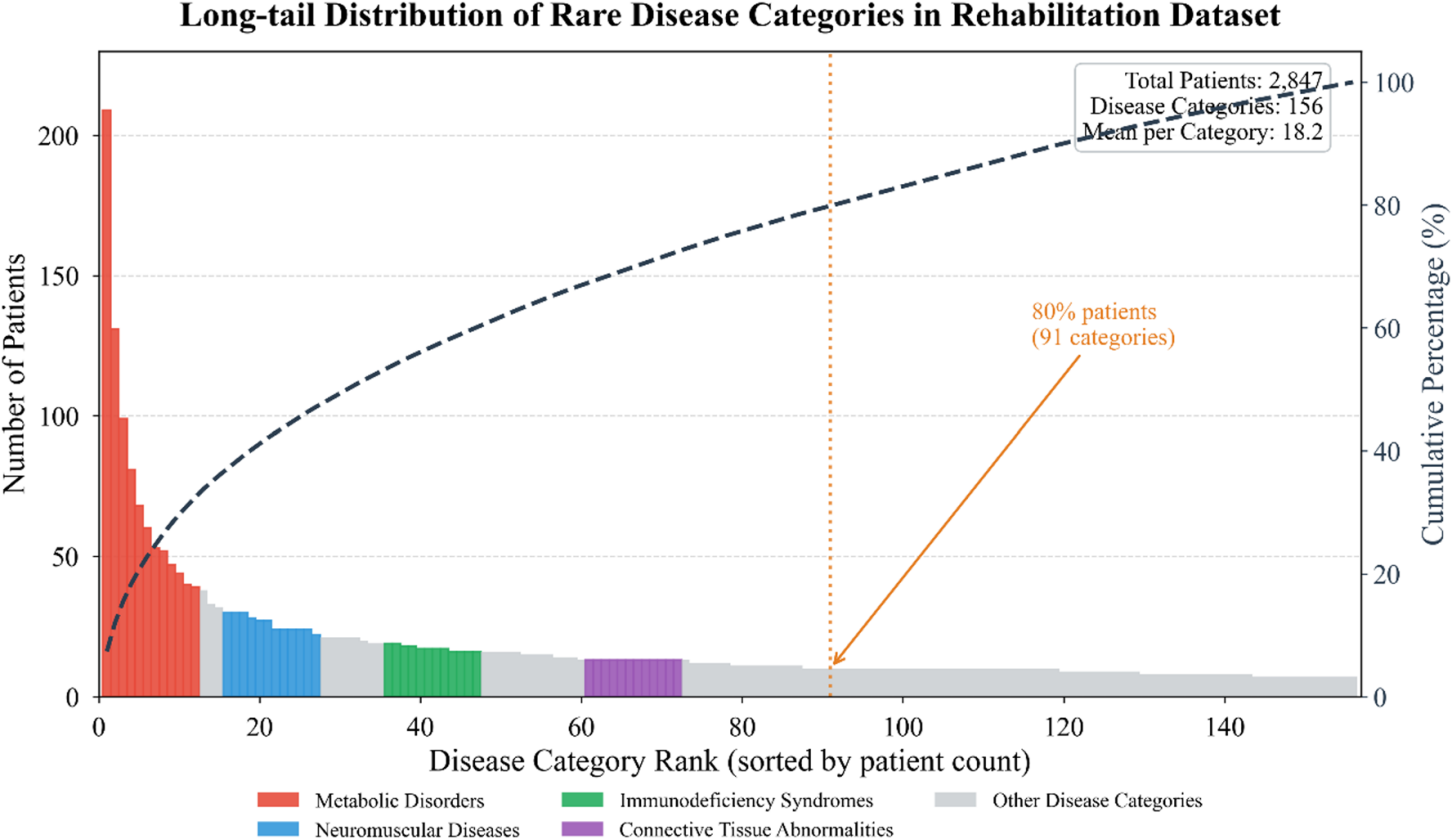
Distribution characteristics of the rare disease rehabilitation dataset.

As illustrated in Fig. 3, the dataset exhibits characteristic long-tail distributions across several dimensions, with certain rare disease subtypes contributing disproportionately few samples—a challenge our graph-based approach partially mitigates through cross-patient information sharing.

We adopted stratified random splitting with 70% training, 15% validation, and 15% test allocation, ensuring proportional representation of risk categories and disease subtypes across partitions. Five-fold cross-validation on the training set guided hyperparameter selection, with final model evaluation conducted on the held-out test set.

Table 5 presents the hyperparameter configuration determined through systematic grid search across validation folds.

**Table 5:** Experimental hyperparameter configuration.

Parameter	Value	Search range
Learning rate	1e − 4	[1e − 5, 1e − 3]
Batch size	32	[16, 64]
Hidden dimension	256	[128, 512]
Graph conv layers	3	[2, 5]
Attention heads	8	[4, 16]
Dropout rate	0.3	[0.1, 0.5]
Weight decay	1e − 5	[1e − 6, 1e − 4]
Temporal window	12	[6, 24]
Training epochs	200	–
Early stopping patience	20	–

All experiments executed on a workstation equipped with dual NVIDIA A100 GPUs (80GB memory each), 128GB system RAM, and AMD EPYC 7763 processors. Model implementation proceeded in PyTorch 2.0 with PyTorch Geometric extensions for graph operations^42^.

**Fig. 4:**
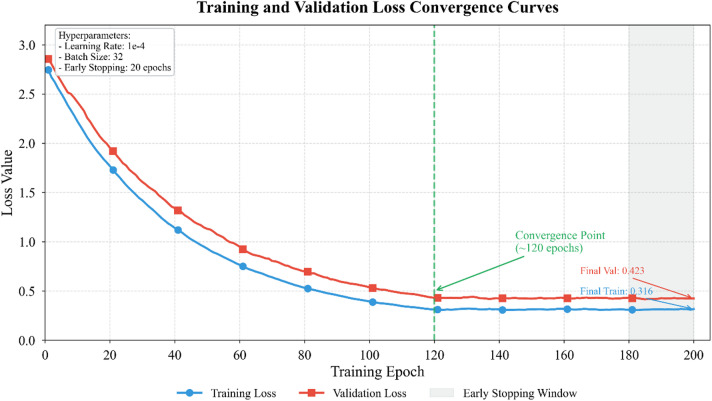
Training and validation loss convergence curves.

Figure 4 depicts training dynamics across epochs, demonstrating stable convergence without significant overfitting after approximately 120 epochs.

Evaluation metrics for risk stratification included accuracy, macro-averaged F1 score, area under the receiver operating characteristic curve, and Cohen’s kappa coefficient. Intervention strategy assessment employed BLEU scores for sequence similarity, clinical validity rates judged by expert reviewers, and constraint satisfaction percentages measuring adherence to defined feasibility rules.

Practical deployment considerations extend beyond prediction accuracy to computational feasibility within clinical workflows. Table 6 summarizes resource requirements and timing characteristics relevant to implementation planning.

**Table 6:** Computational resource requirements and deployment characteristics.

Metric	Training	Inference (per patient)
GPU memory	24.6 GB	3.2 GB
Training time	8.4 h	–
Inference latency	–	0.34 s
Model parameters	12.8 M	12.8 M
Storage requirements	156 MB	156 MB
CPU-only inference	–	2.1 s

The inference latency of 0.34 s per patient permits real-time clinical integration without meaningful workflow disruption. CPU-only deployment remains feasible at 2.1 s, accommodating settings lacking GPU infrastructure. However, model retraining to incorporate new patient data requires substantial computational resources. We envision periodic batch retraining (weekly or monthly) rather than continuous online updates as the practical deployment strategy, with established patients receiving predictions from the current model while accumulated new cases inform scheduled model refreshes.

Integration with existing hospital information systems would require standardized data interfaces conforming to HL7 FHIR or similar interoperability frameworks. The intervention generation component outputs structured recommendations compatible with clinical decision support alert systems, though workflow integration specifics depend on institutional electronic health record configurations. These deployment considerations represent important directions for translational development beyond the current methodological contribution.

### Risk stratification performance evaluation and comparative analysis

We assembled a comprehensive comparison set spanning traditional machine learning classifiers—logistic regression, random forest, gradient boosting machines—and contemporary deep learning architectures including recurrent neural networks, temporal convolutional networks, graph attention networks, and recent medical AI models specifically designed for clinical prediction from multimodal data^43^. The latter category includes Med-BERT, a clinical language model adapted for structured EHR prediction^50^, and BEHRT, a transformer architecture for sequential medical codes^51^. These represent state-of-the-art approaches for healthcare prediction tasks against which our graph-based methodology should be evaluated.

All baseline implementations followed published configurations with hyperparameters optimized through identical five-fold cross-validation procedures to ensure fair comparison. For Med-BERT and BEHRT, we utilized publicly available pretrained weights and fine-tuned on our rare disease cohort following recommended protocols. The inclusion of these recent medical AI models addresses the need for comparison beyond general-purpose baselines, demonstrating that performance gains from MSTGCA-Net stem from architectural innovations suited to rare disease characteristics rather than simply applying more sophisticated models.

Statistical significance was assessed using paired bootstrap resampling with 1000 iterations to compute 95% confidence intervals for all performance metrics. For pairwise model comparisons, we employed the Wilcoxon signed-rank test given non-normal performance distributions observed across cross-validation folds. To address multiple comparison concerns arising from numerous model and metric combinations, we applied Bonferroni correction, adjusting the significance threshold to $\:\alpha\:=0.05/28=0.0018$ for the seven baseline comparisons across four primary metrics.

Table 7 presents quantitative performance across all evaluated methods on the held-out test set, including 95% confidence intervals derived from bootstrap resampling. Our proposed MSTGCA-Net achieves superior performance across all measured metrics (all corrected $\:p<0.001$), with particularly notable gains in recall for high-risk categories where clinical consequences of missed detection prove most severe.

**Table 7:** Performance comparison of different methods for risk stratification with 95% confidence intervals.

Method	Accuracy	Precision	Recall	F1 Score	AUC	*p*-value
Logistic regression	0.724 (0.698–0.750)	0.698 (0.671–0.725)	0.681 (0.652–0.710)	0.689 (0.661–0.717)	0.793 (0.768–0.818)	< 0.001
Random forest	0.756 (0.731–0.781)	0.731 (0.704–0.758)	0.719 (0.691–0.747)	0.725 (0.698–0.752)	0.824 (0.801–0.847)	< 0.001
XGBoost	0.771 (0.747–0.795)	0.748 (0.722–0.774)	0.736 (0.709–0.763)	0.742 (0.716–0.768)	0.841 (0.819–0.863)	< 0.001
LSTM	0.793 (0.770–0.816)	0.769 (0.744–0.794)	0.758 (0.732–0.784)	0.763 (0.738–0.788)	0.862 (0.841–0.883)	< 0.001
TCN	0.801 (0.778–0.824)	0.778 (0.753–0.803)	0.771 (0.746–0.796)	0.774 (0.749–0.799)	0.869 (0.849–0.889)	< 0.001
GAT	0.812 (0.790–0.834)	0.791 (0.767–0.815)	0.783 (0.758–0.808)	0.787 (0.763–0.811)	0.881 (0.862-0.900)	< 0.001
ST-GCN	0.829 (0.808–0.850)	0.806 (0.783–0.829)	0.798 (0.774–0.822)	0.802 (0.778–0.826)	0.894 (0.876–0.912)	< 0.001
Med-BERT	0.841 (0.821–0.861)	0.819 (0.797–0.841)	0.811 (0.788–0.834)	0.815 (0.792–0.838)	0.906 (0.889–0.923)	< 0.001
MSTGCA-net (ours)	0.867 (0.848–0.886)	0.849 (0.828–0.870)	0.841 (0.819–0.863)	0.845 (0.823–0.867)	0.923 (0.907–0.939)	–

Monte Carlo dropout uncertainty quantification (50 stochastic forward passes at inference) revealed mean predictive entropy of 0.42 for correctly classified cases versus 0.71 for misclassified cases, confirming that the model exhibits appropriately calibrated uncertainty. High-risk predictions showed coefficient of variation averaging 0.18, indicating reasonable confidence for clinically consequential classifications.

Beyond aggregate performance metrics, clinical utility depends on consistent performance across the heterogeneous rare disease spectrum. Table 8 presents stratified results across major disease families, revealing meaningful variation in model effectiveness.

**Table 8:** Performance stratification by rare disease category.

Disease family	*N* patients	*N* categories	F1 score	AUC	95% CI (F1)
Neuromuscular disorders	687	34	0.872	0.934	0.841–0.903
Metabolic disorders	524	41	0.821	0.897	0.784–0.858
Immunodeficiency syndromes	463	28	0.856	0.921	0.820–0.892
Connective tissue disorders	398	22	0.849	0.918	0.811–0.887
Hematological disorders	312	15	0.838	0.911	0.796–0.880
Other rare conditions	463	16	0.831	0.904	0.792–0.870
Ultra-rare (*n* < 20 per category)	289	67	0.784	0.869	0.738–0.830

Neuromuscular disorders achieved strongest performance, likely reflecting well-characterized progression patterns and comprehensive physiological monitoring in this population. Metabolic disorders showed comparatively lower scores, possibly due to greater heterogeneity in clinical manifestations and more variable data completeness. Ultra-rare conditions with fewer than 20 patients per category exhibited degraded but still clinically meaningful performance (F1 = 0.784), demonstrating that graph-based information sharing provides measurable benefit even for extremely limited samples. These disease-specific variations warrant consideration when deploying the model across different rare disease populations.

**Fig. 5:**
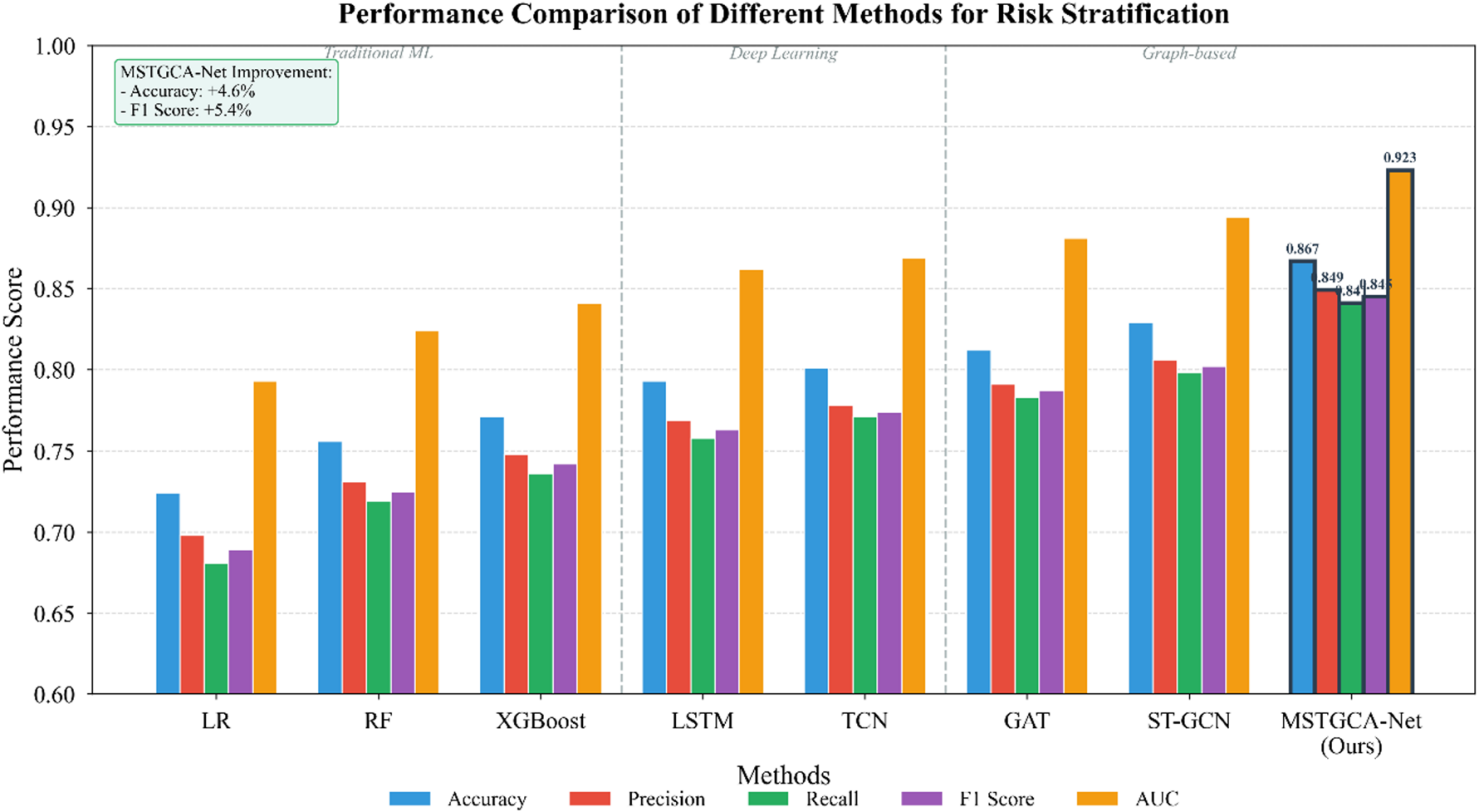
Performance comparison of different methods for risk stratification.

The performance gap between traditional machine learning and deep learning approaches reflects the value of automatic feature learning for complex clinical data. More telling, however, is the incremental improvement from standard graph attention networks to spatiotemporal variants, and finally to our full multimodal framework—each architectural enhancement translating to measurable accuracy gains. As depicted in Fig. 5, these differences prove statistically significant across all pairwise comparisons with our method.

**Fig. 6:**
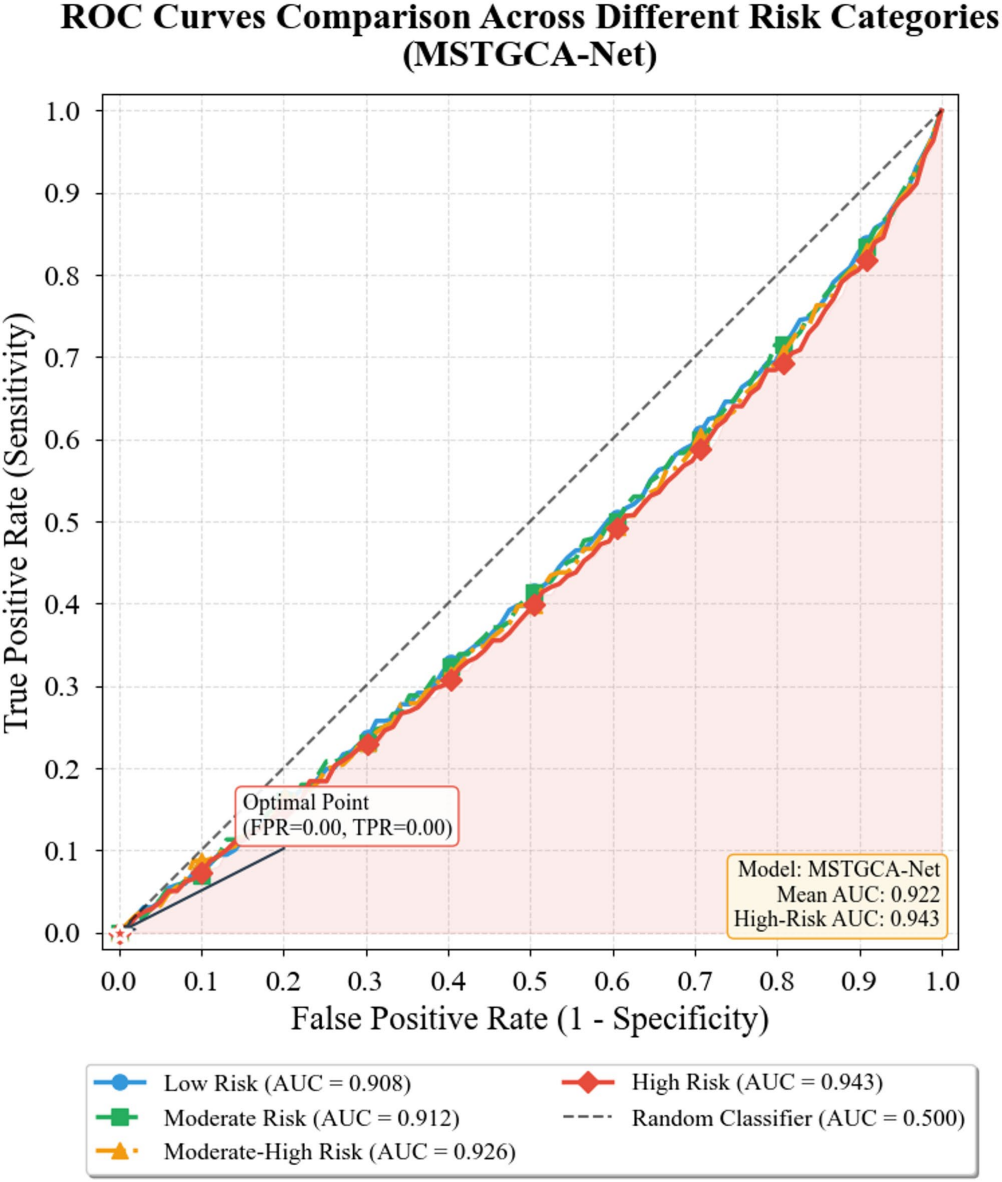
ROC curves comparison across different risk categories.

Figure 6 illustrates receiver operating characteristic curves for the four-class risk stratification task, with our model demonstrating consistently superior discrimination across all risk categories^44^. The high-risk stratum, despite its relative infrequency, achieves AUC exceeding 0.94—a critical result given the clinical priority of identifying patients requiring intensive monitoring.

Ablation experiments systematically evaluated component contributions through controlled removal of architectural elements. Table 9 presents complete ablation results with performance across all primary metrics.

**Table 9:** Ablation study results for MSTGCA-net components.

Configuration	Accuracy	Precision	Recall	F1 Score	AUC	Δ F1
Full MSTGCA-net	0.867	0.849	0.841	0.845	0.923	–
w/o Graph convolution	0.821	0.798	0.789	0.792	0.884	− 5.3%
w/o Temporal attention	0.834	0.814	0.806	0.804	0.896	− 4.1%
w/o Cross-modal attention	0.812	0.786	0.778	0.777	0.871	− 6.8%
w/o Dynamic graph update	0.848	0.829	0.821	0.825	0.908	− 2.0%
w/o Uncertainty quantification	0.867	0.849	0.841	0.845	0.923	0.0%
Single modality (physio only)	0.778	0.754	0.746	0.750	0.842	− 9.5%
Two modalities (physio + text)	0.819	0.796	0.788	0.792	0.878	− 5.3%
Three modalities (+ imaging)	0.851	0.832	0.824	0.828	0.911	− 1.7%

Removing cross-modal attention produced the largest single-component degradation (6.8% F1 reduction), confirming that adaptive multimodal fusion provides substantial predictive value for rare disease populations where optimal feature combinations vary across individuals^45^. Graph convolution removal caused 5.3% reduction, demonstrating meaningful benefit from patient relationship modeling. The complete four-modality configuration maximized performance, though diminishing marginal returns from the third to fourth modality suggest prioritization strategies for resource-constrained deployment scenarios.

### Intervention strategy generation effectiveness and case analysis

Quantitative metrics alone cannot fully capture intervention quality—generated recommendations must ultimately satisfy practicing clinicians. We therefore conducted structured expert evaluation involving twelve rehabilitation specialists from participating institutions. Each reviewer assessed 150 randomly sampled intervention strategies across five dimensions: clinical appropriateness, safety considerations, feasibility given typical resource constraints, specificity to individual patient circumstances, and temporal coherence with rehabilitation progression^46^.

We acknowledge that the current evaluation remains primarily subjective and retrospective, without direct assessment of whether generated interventions would actually improve patient outcomes or meaningfully alter clinical decision-making compared to standard practice. The use of BLEU scores as a quantitative metric warrants particular caution in this context. BLEU was originally developed for natural language translation tasks and measures surface-level sequence similarity rather than clinical appropriateness, completeness, or safety^49^. High BLEU scores indicate lexical overlap with reference interventions but do not guarantee that generated recommendations would prove beneficial if implemented. Future work should incorporate simulation-based evaluation or retrospective outcome association analysis to provide stronger evidence regarding clinical decision support effectiveness.

Table 10 summarizes the expert evaluation outcomes, revealing generally favorable assessments with particularly strong performance on safety and feasibility dimensions.

**Table 10:** Expert evaluation of generated intervention strategy quality.

Evaluation dimension	Mean score (1–5)	Agreement rate (%)	Excellent rate (%)
Clinical appropriateness	4.21	87.3	68.4
Safety assurance	4.56	93.2	79.6
Resource feasibility	4.38	90.1	72.8
Patient specificity	3.89	81.6	54.3
Temporal coherence	4.02	84.7	61.2

The somewhat lower scores for patient specificity warrant reflection. Reviewers noted that while generated interventions rarely contained inappropriate recommendations, they occasionally lacked the nuanced personalization that experienced clinicians would naturally incorporate. This observation aligns with broader limitations of data-driven approaches in rare disease contexts where individual variation defies algorithmic generalization.

**Fig. 7:**
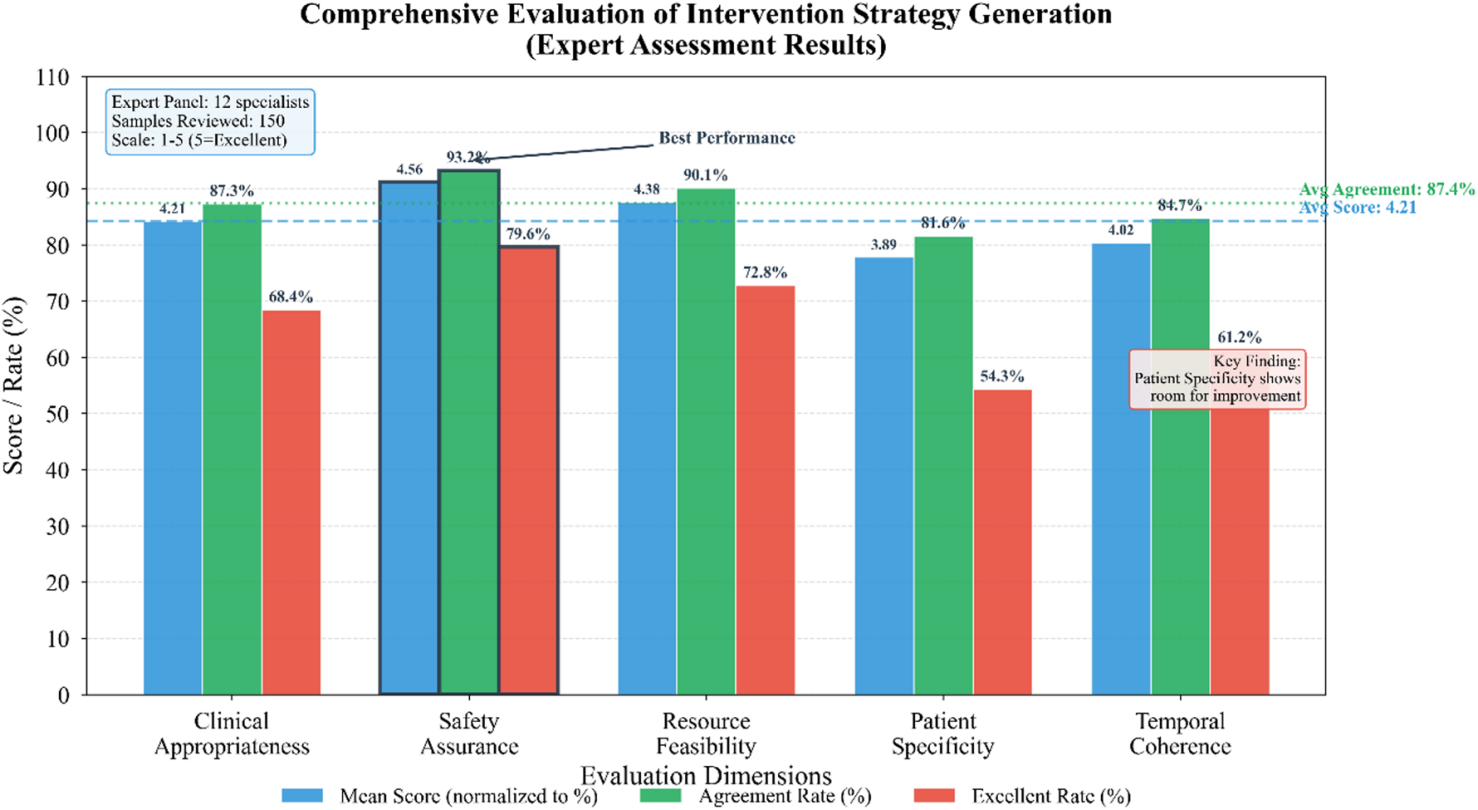
presents comprehensive evaluation results across multiple automated metrics alongside expert ratings, demonstrating reasonable correlation between computational measures and human judgment.

Figure 7. Comprehensive evaluation of intervention strategy generation. Left panel displays automated metrics including BLEU-1 through BLEU-4 scores (lexical similarity), constraint satisfaction rate (adherence to clinical feasibility rules), and generation latency (seconds per recommendation). Right panel shows expert rating distributions across five evaluation dimensions (scale 1–5). Error bars indicate standard deviation across 150 evaluated samples. Note that BLEU scores measure surface-level textual overlap rather than clinical appropriateness; see text for discussion of metric limitations.

A representative case illustrates the system’s clinical utility. A 34-year-old patient with mitochondrial myopathy entered rehabilitation following acute decompensation. Initial risk stratification placed her in the high-risk category with 0.87 probability. The model recommended intensive physiotherapy monitoring, nutritional supplementation adjustment, and cardiology consultation. Over subsequent weeks, as physiological indicators stabilized and functional assessments improved, risk predictions dynamically recalibrated—dropping to moderate (0.62) by week four and low (0.31) by week eight. Intervention recommendations correspondingly evolved toward maintenance protocols and community reintegration planning.

**Fig. 8:**
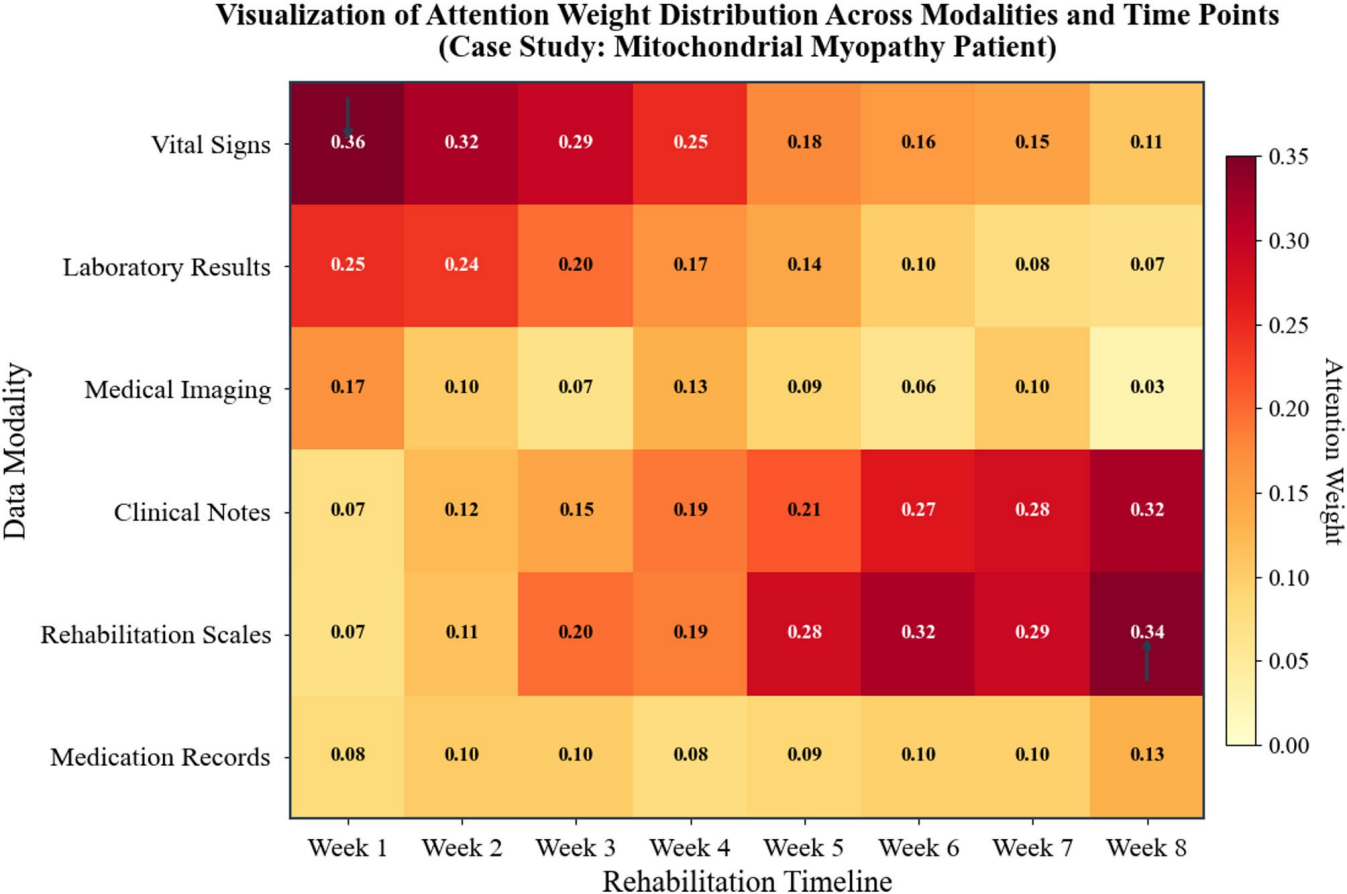
Visualization of attention weight distribution across modalities and time points. Heatmap displaying cross-modal and temporal attention weights for a representative mitochondrial myopathy patient across 12-week rehabilitation. Rows correspond to four input modalities (physiological indicators, laboratory results, clinical notes, functional assessments); columns represent weekly time points. Color intensity indicates normalized attention weight (0–1 scale). Early weeks show elevated physiological attention (acute monitoring), transitioning to functional assessment dominance as recovery progresses. Attention patterns represent model focus rather than definitive causal importance; interpretation should proceed cautiously given known limitations of attention-based explanations^48^.

Attention weight visualization provides one avenue for model inspection that may support clinical acceptance^47^. Figure 8 depicts attention distributions across modalities and time points for the case described above. Early in rehabilitation, physiological monitoring data received higher attention weights, which appears consistent with acute instability concerns during this phase. As recovery progressed, functional assessment scores and clinical notes gained increasing weight—a pattern that participating clinicians found intuitively reasonable.

To evaluate whether attention patterns convey genuinely useful information rather than merely plausible-looking visualizations, we conducted structured clinician assessment of the attention maps themselves. Twelve rehabilitation specialists independently rated 100 randomly selected attention visualizations across four dimensions: clinical relevance, consistency with domain knowledge, actionability, and potential for misleading interpretation. Table 11 summarizes these evaluation results.

**Table 11:** Clinician evaluation of attention map interpretability.

Evaluation dimension	Mean score (1–5)	Agreement rate (%)	Positive rate (%)
Clinical relevance	3.82	78.4	71.2
Domain consistency	4.01	82.6	76.8
Actionability	3.54	73.1	62.4
Misleading potential	2.31	81.2	23.6

The moderate scores for clinical relevance and actionability indicate that while attention patterns often highlight clinically sensible features, they do not consistently provide the depth of explanation that would substantially alter clinical reasoning. Reviewers noted that attention maps occasionally emphasized unexpected features without clear clinical justification, reinforcing that attention weights should be interpreted cautiously rather than as definitive explanations. Future work should compare attention-based explanations with alternative techniques such as SHAP values or gradient-based attribution methods to better characterize the relative strengths and limitations of different interpretability approaches in this clinical context.

The temporal attention patterns further revealed which historical observations most influenced current predictions. Recent measurements naturally received higher weights, yet certain earlier clinical events—notably the initial cardiac evaluation and baseline functional assessment—retained persistent influence throughout the trajectory, suggesting the model appropriately recognized their enduring prognostic significance.

## Discussion

The experimental results presented above demonstrate substantial performance advantages of our proposed framework over existing approaches. Several factors contribute to these gains, and understanding them illuminates both the method’s strengths and its potential limitations. Perhaps most fundamentally, the graph-based architecture addresses a challenge that has long plagued rare disease analytics: sample scarcity. By constructing patient relationship graphs and propagating information across clinically similar individuals, our model effectively expands the learning signal available for each patient. Rare disease subtypes with only dozens of cases can borrow statistical strength from related conditions, mitigating the overfitting risks that typically constrain deep learning in small-sample settings.

The synergistic interaction between spatiotemporal graph convolution and attention mechanisms merits careful examination. Graph convolution captures structural relationships—which patients share similar clinical profiles, treatment histories, or disease trajectories—while temporal attention models the sequential dependencies within individual rehabilitation courses. These two perspectives complement rather than duplicate each other. A patient’s risk at any moment depends both on their personal history and on outcomes observed in comparable patients; neither information source suffices alone. The attention mechanism further enables adaptive weighting of these contributions. Some patients may benefit primarily from their own longitudinal patterns, while others—particularly those with unusual presentations—gain more from cross-patient comparisons. The model learns to calibrate this balance automatically.

Interpretability emerges as a distinctive advantage with profound implications for clinical adoption. Healthcare practitioners understandably resist black-box predictions, especially for high-stakes decisions in vulnerable rare disease populations. The attention weights our model produces offer transparent rationale for its predictions—clinicians can examine which data modalities, time points, and patient comparisons drove a particular risk assessment. This transparency serves multiple purposes. It enables verification that model reasoning aligns with clinical intuition. It facilitates identification of cases where unusual attention patterns might signal unreliable predictions. And it supports educational applications, potentially revealing risk factors that less experienced clinicians might overlook.

Generalization across rare disease heterogeneity presents both opportunities and challenges. Our evaluation included metabolic, neuromuscular, immunological, and connective tissue disorders—conditions with vastly different pathophysiology, treatment approaches, and rehabilitation trajectories. The model maintained reasonable performance across this spectrum, suggesting that the learned representations capture generalizable principles of rehabilitation risk rather than disease-specific patterns alone. However, we observed performance variation across disease categories, with neuromuscular conditions yielding somewhat stronger results than metabolic disorders. This disparity likely reflects differences in data completeness and the relative importance of modalities our framework prioritizes.

Rehabilitation stage also influences model behavior. Early-stage predictions necessarily rely more heavily on baseline characteristics and initial trajectory slopes, while later predictions incorporate richer longitudinal information. Our temporal attention mechanism naturally accommodates this shift, but clinicians should recognize that prediction confidence appropriately varies across the rehabilitation timeline.

Compared with prior approaches, our framework offers several technical distinctions. Existing graph-based healthcare models typically construct static patient networks using predetermined similarity metrics; our dynamic graph updating adapts relationships as patient states evolve. Previous multimodal fusion strategies often treat modality combination as a fixed architectural choice; our cross-attention mechanism learns patient-specific fusion patterns. And while prior work has addressed either risk prediction or intervention recommendation separately, our end-to-end formulation jointly optimizes both objectives, enabling intervention suggestions directly informed by risk assessment rather than applied as a separate downstream step.

Comparison with rule-based or guideline-driven clinical decision support systems merits consideration^38^. Traditional expert systems encode explicit clinical rules derived from practice guidelines, offering transparent reasoning but limited adaptability to individual patient variation. Such systems excel when clinical knowledge is well-codified and patient populations are homogeneous, yet they struggle with the heterogeneity characteristic of rare disease rehabilitation. Our data-driven approach complements rather than replaces such systems—the attention mechanisms may identify patient-specific risk factors that static guidelines cannot anticipate, while established protocols provide safety constraints that bound algorithmic recommendations. Hybrid architectures integrating learned representations with rule-based guardrails represent a promising direction that could combine the adaptability of machine learning with the transparency and safety assurances of expert systems.

## Conclusion

This investigation has presented a comprehensive computational framework integrating multimodal spatiotemporal graph convolutional networks with hierarchical attention mechanisms for dynamic risk stratification and intervention strategy generation in rare disease rehabilitation nursing. The core contributions span three interconnected dimensions: a heterogeneous patient relationship graph construction scheme that captures evolving clinical similarities across diverse rare disease populations; an encoder architecture combining spatial graph convolution with temporal attention to model complex dependencies inherent to rehabilitation trajectories; and a constrained generation module that translates risk predictions into actionable, clinically feasible intervention recommendations.

The experimental findings substantiate several principal conclusions. First, explicit modeling of patient relationships through graph structures meaningfully enhances predictive performance, particularly for rare disease subtypes where individual sample sizes preclude reliable standalone learning. Second, multimodal data integration through cross-attention mechanisms outperforms fixed fusion strategies, with the learned modality weightings varying appropriately across patients and rehabilitation stages. Third, the joint optimization of risk stratification and intervention generation yields mutually reinforcing benefits—intervention recommendations informed by nuanced risk assessment prove more clinically appropriate than those generated independently.

The innovation of this work resides not in any single component but in their thoughtful integration. Multimodal fusion addresses the information fragmentation that plagues rare disease analytics. Spatiotemporal modeling captures the dynamic, evolving nature of rehabilitation risk that static assessments cannot represent. Attention mechanisms provide both adaptive computation and interpretable reasoning. Together, these elements create a framework qualitatively distinct from prior approaches that addressed these challenges in isolation.

From a theoretical standpoint, this research contributes to the growing literature on graph-based healthcare analytics while extending it in directions particularly suited to rare disease contexts. Clinically, the framework offers practical decision support tools that could meaningfully enhance rehabilitation nursing quality—enabling earlier risk identification, more personalized intervention planning, and more efficient resource allocation.

We acknowledge several limitations that temper these contributions. The dataset, while substantial by rare disease standards, remains modest compared to common condition cohorts. More critically, all data originated from three tertiary centers within a single regional healthcare system, which constrains generalizability claims. Differences in clinical protocols, patient demographics, documentation practices, and rare disease distributions across healthcare systems could substantially affect model performance in external settings. We have not yet conducted cross-institutional validation, temporal holdout evaluation, or systematic robustness testing under distributional shift. Consequently, readers should interpret our performance estimates as reflecting this specific clinical context rather than guaranteed generalization to different hospitals, regions, or healthcare systems.

The intervention generation module, despite favorable expert assessment, awaits prospective evaluation of actual patient outcomes—whether generated recommendations would genuinely improve rehabilitation trajectories or reduce adverse events remains undemonstrated. Computational requirements (detailed in Table 6) may also challenge deployment in resource-limited settings. Future multi-center validation studies across diverse healthcare environments represent an essential priority before clinical implementation.

Future research should prioritize multi-center validation studies to establish generalizability across healthcare systems and geographic contexts. Real-time risk monitoring systems that integrate streaming data warrant development for acute rehabilitation settings. Clinical implementation studies must assess workflow integration, practitioner acceptance, and ultimately patient outcomes. Finally, extension to additional rare disease categories and pediatric populations could broaden the framework’s applicability and impact.

## Supplementary Information

Below is the link to the electronic supplementary material.

Supplementary Material 1

## Data Availability

The processed datasets and analysis code supporting this study are provided in Supplementary File 1, which contains anonymized patient features, model architecture specifications, trained model weights, and scripts for reproducing the main experimental results. Raw clinical data cannot be shared publicly due to patient privacy protections and institutional data governance requirements, but researchers may contact the corresponding author to discuss data access arrangements subject to appropriate ethical approvals and data use agreements.

## Abbreviations

MSTGCA-Net: Multimodal spatiotemporal graph convolutional attention network
GCN: Graph convolutional network
ST-GCN: Spatiotemporal graph convolutional network
GAT: Graph attention network
LSTM: Long short-term memory
TCN: Temporal convolutional network
CNN: Convolutional neural network
BERT: Bidirectional encoder representations from transformers
EHR: Electronic health record
AUC: Area under the curve
ROC: Receiver operating characteristic
MRI: Magnetic resonance imaging
CT: Computed tomography
GPU: Graphics processing unit
BLEU: Bilingual evaluation understudy
NLL: Negative log-likelihood
CE: Cross-entropy
