# Supplementary material for: Multimodal spatiotemporal graph convolutional attention network for dynamic risk stratification and intervention strategy generation in rare disease rehabilitation nursing
Source: Sci Rep. 2026 Jan 30;16:6758. doi: 10.1038/s41598-026-37095-9 (PMC12913661; doi:10.1038/s41598-026-37095-9)
Supplement: Supplementary file 1 — Supplementary Material 1 [file 41598_2026_37095_MOESM1_ESM.docx]

**Supplementary File 1: Supplementary Materials for MSTGCA-Net**

**Supplementary Table S1. Complete Disease Category Distribution Across Data Splits**

| **Disease Family** | **Disease Category** | **ICD-10 Code** | **Training (n)** | **Validation (n)** | **Test (n)** | **Total (n)** |
| --- | --- | --- | --- | --- | --- | --- |
| Neuromuscular | Duchenne Muscular Dystrophy | G71.01 | 89 | 19 | 18 | 126 |
| Neuromuscular | Spinal Muscular Atrophy Type II | G12.1 | 76 | 16 | 17 | 109 |
| Neuromuscular | Myasthenia Gravis | G70.0 | 68 | 15 | 14 | 97 |
| Neuromuscular | Charcot-Marie-Tooth Disease | G60.0 | 54 | 12 | 11 | 77 |
| Neuromuscular | Becker Muscular Dystrophy | G71.02 | 42 | 9 | 9 | 60 |
| Neuromuscular | Limb-Girdle Muscular Dystrophy | G71.09 | 38 | 8 | 8 | 54 |
| Neuromuscular | Facioscapulohumeral Dystrophy | G71.02 | 31 | 7 | 7 | 45 |
| Neuromuscular | Pompe Disease | E74.02 | 28 | 6 | 6 | 40 |
| Neuromuscular | Friedreich Ataxia | G11.11 | 24 | 5 | 5 | 34 |
| Neuromuscular | Other Neuromuscular (n<30) | Various | 31 | 7 | 7 | 45 |
| Metabolic | Phenylketonuria | E70.0 | 72 | 15 | 16 | 103 |
| Metabolic | Gaucher Disease | E75.22 | 58 | 12 | 13 | 83 |
| Metabolic | Fabry Disease | E75.21 | 49 | 11 | 10 | 70 |
| Metabolic | Mucopolysaccharidosis Type I | E76.01 | 41 | 9 | 9 | 59 |
| Metabolic | Mucopolysaccharidosis Type II | E76.1 | 36 | 8 | 7 | 51 |
| Metabolic | Niemann-Pick Disease | E75.24 | 29 | 6 | 6 | 41 |
| Metabolic | Maple Syrup Urine Disease | E71.0 | 24 | 5 | 5 | 34 |
| Metabolic | Homocystinuria | E72.11 | 21 | 5 | 4 | 30 |
| Metabolic | Other Metabolic (n<30) | Various | 38 | 8 | 7 | 53 |
| Immunodeficiency | Common Variable Immunodeficiency | D83.9 | 67 | 14 | 15 | 96 |
| Immunodeficiency | X-linked Agammaglobulinemia | D80.0 | 52 | 11 | 11 | 74 |
| Immunodeficiency | Severe Combined Immunodeficiency | D81.9 | 44 | 9 | 10 | 63 |
| Immunodeficiency | Wiskott-Aldrich Syndrome | D82.0 | 38 | 8 | 8 | 54 |
| Immunodeficiency | Chronic Granulomatous Disease | D71 | 31 | 7 | 6 | 44 |
| Immunodeficiency | Other Immunodeficiency (n<30) | Various | 92 | 20 | 20 | 132 |
| Connective Tissue | Ehlers-Danlos Syndrome | Q79.6 | 61 | 13 | 13 | 87 |
| Connective Tissue | Marfan Syndrome | Q87.40 | 53 | 11 | 12 | 76 |
| Connective Tissue | Osteogenesis Imperfecta | Q78.0 | 47 | 10 | 10 | 67 |
| Connective Tissue | Loeys-Dietz Syndrome | Q87.41 | 34 | 7 | 7 | 48 |
| Connective Tissue | Other Connective Tissue (n<30) | Various | 84 | 18 | 18 | 120 |
| Hematological | Hereditary Spherocytosis | D58.0 | 48 | 10 | 10 | 68 |
| Hematological | Fanconi Anemia | D61.09 | 41 | 9 | 9 | 59 |
| Hematological | Diamond-Blackfan Anemia | D61.01 | 35 | 7 | 8 | 50 |
| Hematological | Severe Congenital Neutropenia | D70.0 | 29 | 6 | 6 | 41 |
| Hematological | Other Hematological (n<30) | Various | 66 | 14 | 14 | 94 |
| Other | Prader-Willi Syndrome | Q87.11 | 38 | 8 | 8 | 54 |
| Other | Angelman Syndrome | Q93.51 | 32 | 7 | 7 | 46 |
| Other | Rett Syndrome | F84.2 | 28 | 6 | 6 | 40 |
| Other | Tuberous Sclerosis Complex | Q85.1 | 25 | 5 | 6 | 36 |
| Other | Other Rare Conditions (n<30) | Various | 201 | 43 | 43 | 287 |

**Supplementary Table S2. Missing Data Patterns by Data Modality and Disease Family**

| **Disease Family** | **Vital Signs (%)** | **Laboratory (%)** | **Imaging (%)** | **Clinical Notes (%)** | **Rehab Scales (%)** | **Overall (%)** |
| --- | --- | --- | --- | --- | --- | --- |
| Neuromuscular | 4.2 | 8.7 | 12.3 | 3.1 | 5.8 | 6.8 |
| Metabolic | 5.1 | 14.6 | 18.9 | 4.8 | 7.2 | 10.1 |
| Immunodeficiency | 3.8 | 11.2 | 15.6 | 2.9 | 6.4 | 8.0 |
| Connective Tissue | 4.5 | 9.8 | 14.1 | 3.4 | 6.1 | 7.6 |
| Hematological | 3.6 | 7.4 | 16.8 | 2.7 | 5.9 | 7.3 |
| Other | 6.8 | 16.3 | 21.4 | 5.6 | 8.9 | 11.8 |
| **Overall** | **4.7** | **11.3** | **16.5** | **3.8** | **6.7** | **8.6** |

**Supplementary Table S3. Imputation Method Comparison on Validation Subset**

| **Imputation Strategy** | **Accuracy** | **F1 Score** | **AUC** | **Δ F1 vs. Disease-Stratified** |
| --- | --- | --- | --- | --- |
| Disease-Stratified Median | 0.851 | 0.832 | 0.911 | — |
| Global Median | 0.834 | 0.814 | 0.896 | -1.8% |
| MICE (5 iterations) | 0.847 | 0.828 | 0.908 | -0.4% |
| MICE (10 iterations) | 0.849 | 0.830 | 0.909 | -0.2% |
| Last Observation Carried Forward | 0.841 | 0.821 | 0.901 | -1.1% |
| K-Nearest Neighbors (k=5) | 0.839 | 0.819 | 0.899 | -1.3% |
| Mean Imputation | 0.828 | 0.808 | 0.889 | -2.4% |

**Supplementary Table S4. Disease-Specific Imputation Effects on Model Performance**

| **Disease Family** | **Global Median F1** | **Disease-Stratified F1** | **Improvement (%)** | **p-value** |
| --- | --- | --- | --- | --- |
| Neuromuscular | 0.861 | 0.872 | +1.3% | 0.024 |
| Metabolic | 0.801 | 0.821 | +2.5% | 0.008 |
| Immunodeficiency | 0.849 | 0.856 | +0.8% | 0.112 |
| Connective Tissue | 0.841 | 0.849 | +1.0% | 0.067 |
| Hematological | 0.832 | 0.838 | +0.7% | 0.134 |
| Other | 0.812 | 0.831 | +2.3% | 0.011 |

**Supplementary Table S5. Detailed Feature Description for Each Modality**

| **Modality** | **Feature Name** | **Description** | **Unit** | **Range** |
| --- | --- | --- | --- | --- |
| Vital Signs | Heart Rate | Resting heart rate | bpm | 40-180 |
| Vital Signs | Heart Rate Variability | SDNN of RR intervals | ms | 10-200 |
| Vital Signs | Systolic BP | Systolic blood pressure | mmHg | 70-200 |
| Vital Signs | Diastolic BP | Diastolic blood pressure | mmHg | 40-120 |
| Vital Signs | Respiratory Rate | Breaths per minute | /min | 8-40 |
| Vital Signs | SpO2 | Oxygen saturation | % | 80-100 |
| Vital Signs | Temperature | Body temperature | °C | 35-40 |
| Vital Signs | Pain Score | Numeric pain rating | - | 0-10 |
| Vital Signs | Fatigue Level | Subjective fatigue rating | - | 0-10 |
| Vital Signs | Sleep Quality | Pittsburgh Sleep Quality Index | - | 0-21 |
| Vital Signs | Activity Level | Daily step count (normalized) | - | 0-1 |
| Vital Signs | Weight Change | Weekly weight change | kg | -5 to +5 |
| Laboratory | Hemoglobin | Blood hemoglobin level | g/dL | 6-18 |
| Laboratory | WBC Count | White blood cell count | ×10³/μL | 1-30 |
| Laboratory | Platelet Count | Platelet count | ×10³/μL | 50-500 |
| Laboratory | Creatinine | Serum creatinine | mg/dL | 0.2-5.0 |
| Laboratory | BUN | Blood urea nitrogen | mg/dL | 5-80 |
| Laboratory | ALT | Alanine aminotransferase | U/L | 5-200 |
| Laboratory | AST | Aspartate aminotransferase | U/L | 5-200 |
| Laboratory | Albumin | Serum albumin | g/dL | 2-5 |
| Laboratory | CRP | C-reactive protein | mg/L | 0-100 |
| Laboratory | ESR | Erythrocyte sedimentation rate | mm/hr | 0-100 |
| Laboratory | Creatine Kinase | Serum creatine kinase | U/L | 20-5000 |
| Laboratory | Lactate | Blood lactate level | mmol/L | 0.5-10 |
| Laboratory | ... (36 additional) | Disease-specific markers | Various | Various |
| Imaging | CNN Feature 1-512 | ResNet-50 extracted features | - | Continuous |
| Clinical Notes | BERT Embedding 1-768 | ClinicalBERT embeddings | - | Continuous |
| Rehab Scales | FIM Motor Score | Functional Independence Measure | - | 13-91 |
| Rehab Scales | FIM Cognitive Score | FIM cognitive subscale | - | 5-35 |
| Rehab Scales | Barthel Index | Activities of daily living | - | 0-100 |
| Rehab Scales | 6MWT Distance | Six-minute walk test | meters | 0-800 |
| Rehab Scales | Berg Balance Score | Balance assessment | - | 0-56 |
| Rehab Scales | MRC Strength Scale | Muscle strength (composite) | - | 0-60 |
| Rehab Scales | PROMIS Fatigue | Patient-reported fatigue | T-score | 20-80 |
| Rehab Scales | SF-36 Physical | Quality of life physical | - | 0-100 |
| Rehab Scales | ... (16 additional) | Condition-specific scales | Various | Various |

**Supplementary Table S6. Intervention Vocabulary and Constraint Matrix Summary**

| **Intervention Category** | **Number of Actions** | **Example Actions** |
| --- | --- | --- |
| Medication Adjustment | 28 | Increase analgesic dose, Add muscle relaxant, Reduce immunosuppressant |
| Therapy Intensity | 18 | Increase PT frequency, Add occupational therapy, Reduce exercise intensity |
| Monitoring | 15 | Increase vital sign frequency, Add cardiac monitoring, Weekly labs |
| Consultation | 12 | Cardiology referral, Pulmonology consult, Genetics evaluation |
| Nutrition | 11 | Increase protein intake, Add supplements, Modify diet texture |
| Psychosocial | 9 | Add counseling, Family meeting, Support group referral |
| Equipment | 8 | Wheelchair assessment, Orthotics fitting, Home modification |
| Discharge Planning | 7 | Community services, Home health setup, Follow-up scheduling |
| **Total** | **108** | — |

**Supplementary Table S7. Pairwise Constraint Matrix Examples (Partial)**

| **Intervention A** | **Intervention B** | **Constraint** | **Rationale** |
| --- | --- | --- | --- |
| Increase exercise intensity | Reduce activity level | Incompatible | Contradictory actions |
| Add anticoagulant | Increase PT intensity | Conditional | Risk of bleeding |
| Reduce immunosuppressant | Increase infection monitoring | Required pair | Safety monitoring |
| Discharge planning | ICU transfer | Incompatible | Contradictory care level |
| Add opioid analgesic | Driving clearance | Incompatible | Safety concern |
| Increase oral intake | NPO status | Incompatible | Contradictory |
| Cardiac rehabilitation | Unstable angina status | Conditional | Requires stabilization |
| Weight-bearing exercise | Non-weight bearing order | Incompatible | Contradictory |

**Supplementary Table S8. Cross-Validation Results Across Five Folds**

| **Fold** | **Accuracy** | **Precision** | **Recall** | **F1 Score** | **AUC** | **Training Time (hr)** |
| --- | --- | --- | --- | --- | --- | --- |
| 1 | 0.861 | 0.843 | 0.836 | 0.839 | 0.918 | 8.2 |
| 2 | 0.869 | 0.851 | 0.844 | 0.847 | 0.925 | 8.5 |
| 3 | 0.864 | 0.846 | 0.838 | 0.842 | 0.921 | 8.3 |
| 4 | 0.871 | 0.854 | 0.846 | 0.850 | 0.927 | 8.6 |
| 5 | 0.866 | 0.849 | 0.841 | 0.845 | 0.923 | 8.4 |
| **Mean ± SD** | **0.866 ± 0.004** | **0.849 ± 0.004** | **0.841 ± 0.004** | **0.845 ± 0.004** | **0.923 ± 0.003** | **8.4 ± 0.2** |

**Supplementary Table S9. Per-Class Performance Metrics**

| **Risk Category** | **Precision** | **Recall** | **F1 Score** | **Support (Test)** | **AUC (One-vs-Rest)** |
| --- | --- | --- | --- | --- | --- |
| High Risk | 0.891 | 0.876 | 0.883 | 80 | 0.941 |
| Moderate-High Risk | 0.834 | 0.821 | 0.827 | 104 | 0.912 |
| Moderate-Low Risk | 0.829 | 0.836 | 0.832 | 131 | 0.908 |
| Low Risk | 0.842 | 0.831 | 0.836 | 112 | 0.921 |
| **Macro Average** | **0.849** | **0.841** | **0.845** | **427** | **0.923** |

**Supplementary Table S10. Confusion Matrix for Risk Stratification (Test Set)**

| **True \ Predicted** | **High Risk** | **Moderate-High** | **Moderate-Low** | **Low Risk** |
| --- | --- | --- | --- | --- |
| High Risk | 70 | 6 | 3 | 1 |
| Moderate-High | 5 | 85 | 11 | 3 |
| Moderate-Low | 2 | 9 | 110 | 10 |
| Low Risk | 1 | 2 | 16 | 93 |

**Supplementary Table S11. Hyperparameter Sensitivity Analysis**

| **Parameter** | **Value Range Tested** | **Optimal Value** | **F1 at Optimal** | **F1 Range** |
| --- | --- | --- | --- | --- |
| Learning Rate | 1e-5, 5e-5, 1e-4, 5e-4, 1e-3 | 1e-4 | 0.845 | 0.798-0.845 |
| Batch Size | 16, 32, 64, 128 | 32 | 0.845 | 0.831-0.845 |
| Hidden Dimension | 128, 256, 512, 768 | 256 | 0.845 | 0.824-0.845 |
| Graph Conv Layers | 2, 3, 4, 5 | 3 | 0.845 | 0.827-0.845 |
| Attention Heads | 4, 8, 12, 16 | 8 | 0.845 | 0.836-0.845 |
| Dropout Rate | 0.1, 0.2, 0.3, 0.4, 0.5 | 0.3 | 0.845 | 0.821-0.845 |
| Weight Decay | 1e-6, 1e-5, 1e-4, 1e-3 | 1e-5 | 0.845 | 0.829-0.845 |
| Temporal Window | 6, 12, 18, 24 | 12 | 0.845 | 0.831-0.845 |

**Supplementary Table S12. Expert Reviewer Demographics and Qualifications**

| **Reviewer ID** | **Specialty** | **Years Experience** | **Institution** | **Rare Disease Expertise** |
| --- | --- | --- | --- | --- |
| R1 | Rehabilitation Medicine | 18 | Center A | Neuromuscular |
| R2 | Rehabilitation Nursing | 15 | Center A | General |
| R3 | Physical Therapy | 12 | Center A | Metabolic |
| R4 | Rehabilitation Medicine | 22 | Center B | Immunodeficiency |
| R5 | Rehabilitation Nursing | 14 | Center B | Connective Tissue |
| R6 | Occupational Therapy | 10 | Center B | General |
| R7 | Rehabilitation Medicine | 16 | Center C | Neuromuscular |
| R8 | Rehabilitation Nursing | 13 | Center C | Metabolic |
| R9 | Physical Therapy | 11 | Center C | General |
| R10 | Clinical Pharmacist | 9 | Center A | Medication Management |
| R11 | Social Work | 8 | Center B | Psychosocial |
| R12 | Rehabilitation Medicine | 20 | Center C | Hematological |

**Supplementary Table S13. Inter-Rater Reliability Statistics**

| **Evaluation Dimension** | **Fleiss' Kappa** | **ICC (2,k)** | **95% CI** |
| --- | --- | --- | --- |
| Clinical Appropriateness | 0.72 | 0.84 | 0.79-0.88 |
| Safety Assurance | 0.81 | 0.91 | 0.87-0.94 |
| Resource Feasibility | 0.76 | 0.87 | 0.83-0.91 |
| Patient Specificity | 0.64 | 0.78 | 0.72-0.83 |
| Temporal Coherence | 0.69 | 0.81 | 0.76-0.86 |

**Supplementary Table S14. Automated Intervention Generation Metrics**

| **Metric** | **Score** | **95% CI** |
| --- | --- | --- |
| BLEU-1 | 0.684 | 0.661-0.707 |
| BLEU-2 | 0.571 | 0.546-0.596 |
| BLEU-3 | 0.483 | 0.456-0.510 |
| BLEU-4 | 0.412 | 0.384-0.440 |
| ROUGE-L | 0.592 | 0.568-0.616 |
| Constraint Satisfaction Rate | 0.961 | 0.948-0.974 |
| Average Generation Length | 4.7 actions | 4.2-5.2 |
| Generation Latency | 0.12 sec | 0.09-0.15 |

**Supplementary Table S15. Comparison with Alternative Interpretability Methods**

| **Method** | **Computation Time (ms)** | **Clinician Relevance Score** | **Consistency Score** |
| --- | --- | --- | --- |
| Attention Weights (Ours) | 12 | 3.82 | 0.78 |
| SHAP Values | 2840 | 3.91 | 0.82 |
| Integrated Gradients | 1560 | 3.76 | 0.75 |
| LIME | 4210 | 3.68 | 0.71 |
| Grad-CAM (for imaging) | 89 | 3.54 | 0.69 |

**Supplementary Code S1. Model Architecture Implementation**

import torch

import torch.nn as nn

import torch.nn.functional as F

from torch_geometric.nn import GCNConv, GATConv

from torch_geometric.data import Data, Batch

class ModalityEncoder(nn.Module):

"""Modality-specific encoder for different data types."""

def __init__(self, input_dim, hidden_dim, modality_type='numeric'):

super(ModalityEncoder, self).__init__()

self.modality_type = modality_type

if modality_type == 'numeric':

self.encoder = nn.Sequential(

nn.Linear(input_dim, hidden_dim),

nn.LayerNorm(hidden_dim),

nn.ReLU(),

nn.Dropout(0.3),

nn.Linear(hidden_dim, hidden_dim)

)

elif modality_type == 'text':

self.encoder = nn.Sequential(

nn.Linear(input_dim, hidden_dim * 2),

nn.LayerNorm(hidden_dim * 2),

nn.GELU(),

nn.Dropout(0.3),

nn.Linear(hidden_dim * 2, hidden_dim)

)

elif modality_type == 'imaging':

self.encoder = nn.Sequential(

nn.Linear(input_dim, hidden_dim * 2),

nn.LayerNorm(hidden_dim * 2),

nn.ReLU(),

nn.Dropout(0.3),

nn.Linear(hidden_dim * 2, hidden_dim)

)

def forward(self, x):

return self.encoder(x)

class CrossModalAttention(nn.Module):

"""Cross-modal attention mechanism for multimodal fusion."""

def __init__(self, hidden_dim, num_heads=8):

super(CrossModalAttention, self).__init__()

self.num_heads = num_heads

self.head_dim = hidden_dim // num_heads

self.scale = self.head_dim ** -0.5

self.q_proj = nn.Linear(hidden_dim, hidden_dim)

self.k_proj = nn.Linear(hidden_dim, hidden_dim)

self.v_proj = nn.Linear(hidden_dim, hidden_dim)

self.out_proj = nn.Linear(hidden_dim, hidden_dim)

self.dropout = nn.Dropout(0.1)

self.layer_norm = nn.LayerNorm(hidden_dim)

def forward(self, query_modality, key_modality, value_modality):

batch_size = query_modality.size(0)

Q = self.q_proj(query_modality).view(batch_size, -1, self.num_heads, self.head_dim).transpose(1, 2)

K = self.k_proj(key_modality).view(batch_size, -1, self.num_heads, self.head_dim).transpose(1, 2)

V = self.v_proj(value_modality).view(batch_size, -1, self.num_heads, self.head_dim).transpose(1, 2)

attn_weights = torch.matmul(Q, K.transpose(-2, -1)) * self.scale

attn_weights = F.softmax(attn_weights, dim=-1)

attn_weights = self.dropout(attn_weights)

attn_output = torch.matmul(attn_weights, V)

attn_output = attn_output.transpose(1, 2).contiguous().view(batch_size, -1, self.num_heads * self.head_dim)

output = self.out_proj(attn_output)

output = self.layer_norm(output + query_modality)

return output, attn_weights

class AdaptiveModalityFusion(nn.Module):

"""Adaptive fusion of multiple modalities with learnable weights."""

def __init__(self, hidden_dim, num_modalities=4, num_heads=8):

super(AdaptiveModalityFusion, self).__init__()

self.num_modalities = num_modalities

self.modality_weights = nn.Parameter(torch.ones(num_modalities) / num_modalities)

self.cross_attention = CrossModalAttention(hidden_dim, num_heads)

self.fusion_gate = nn.Sequential(

nn.Linear(hidden_dim * num_modalities, hidden_dim),

nn.Sigmoid()

)

self.output_proj = nn.Linear(hidden_dim * num_modalities, hidden_dim)

def forward(self, modality_features):

# modality_features: list of [batch_size, hidden_dim] tensors

weights = F.softmax(self.modality_weights, dim=0)

weighted_features = []

for i, feat in enumerate(modality_features):

weighted_features.append(weights[i] * feat)

# Cross-modal attention between all pairs

attended_features = []

for i in range(self.num_modalities):

cross_attended = modality_features[i]

for j in range(self.num_modalities):

if i != j:

attended, _ = self.cross_attention(

modality_features[i].unsqueeze(1),

modality_features[j].unsqueeze(1),

modality_features[j].unsqueeze(1)

)

cross_attended = cross_attended + attended.squeeze(1)

attended_features.append(cross_attended)

# Concatenate and apply gating

concat_features = torch.cat(attended_features, dim=-1)

gate = self.fusion_gate(concat_features)

fused = self.output_proj(concat_features)

fused = gate * fused + (1 - gate) * sum(weighted_features)

return fused, weights

class GatedGraphConvolution(nn.Module):

"""Gated graph convolution layer with selective message passing."""

def __init__(self, in_channels, out_channels):

super(GatedGraphConvolution, self).__init__()

self.conv = GCNConv(in_channels, out_channels)

self.gate_conv = GCNConv(in_channels, out_channels)

self.layer_norm = nn.LayerNorm(out_channels)

def forward(self, x, edge_index, edge_weight=None):

h = self.conv(x, edge_index, edge_weight)

g = torch.sigmoid(self.gate_conv(x, edge_index, edge_weight))

output = g * torch.tanh(h)

output = self.layer_norm(output + x if x.size(-1) == output.size(-1) else output)

return output

class SpatialGraphEncoder(nn.Module):

"""Spatial encoder using gated graph convolutions."""

def __init__(self, input_dim, hidden_dim, num_layers=3):

super(SpatialGraphEncoder, self).__init__()

self.layers = nn.ModuleList()

self.input_proj = nn.Linear(input_dim, hidden_dim)

for _ in range(num_layers):

self.layers.append(GatedGraphConvolution(hidden_dim, hidden_dim))

self.output_proj = nn.Linear(hidden_dim, hidden_dim)

def forward(self, x, edge_index, edge_weight=None):

x = self.input_proj(x)

for layer in self.layers:

x = layer(x, edge_index, edge_weight)

return self.output_proj(x)

class TemporalAttentionEncoder(nn.Module):

"""Temporal attention encoder with relative position encoding."""

def __init__(self, hidden_dim, num_heads=8, max_seq_len=24):

super(TemporalAttentionEncoder, self).__init__()

self.hidden_dim = hidden_dim

self.num_heads = num_heads

self.head_dim = hidden_dim // num_heads

self.q_proj = nn.Linear(hidden_dim, hidden_dim)

self.k_proj = nn.Linear(hidden_dim, hidden_dim)

self.v_proj = nn.Linear(hidden_dim, hidden_dim)

self.out_proj = nn.Linear(hidden_dim, hidden_dim)

# Relative position encoding

self.rel_pos_embedding = nn.Embedding(2 * max_seq_len - 1, num_heads)

self.dropout = nn.Dropout(0.1)

self.layer_norm = nn.LayerNorm(hidden_dim)

def forward(self, x, mask=None):

# x: [batch_size, seq_len, hidden_dim]

batch_size, seq_len, _ = x.size()

Q = self.q_proj(x).view(batch_size, seq_len, self.num_heads, self.head_dim).transpose(1, 2)

K = self.k_proj(x).view(batch_size, seq_len, self.num_heads, self.head_dim).transpose(1, 2)

V = self.v_proj(x).view(batch_size, seq_len, self.num_heads, self.head_dim).transpose(1, 2)

# Compute attention scores

attn_scores = torch.matmul(Q, K.transpose(-2, -1)) / (self.head_dim ** 0.5)

# Add relative position bias

positions = torch.arange(seq_len, device=x.device)

rel_pos = positions.unsqueeze(0) - positions.unsqueeze(1) + seq_len - 1

rel_pos_bias = self.rel_pos_embedding(rel_pos).permute(2, 0, 1)

attn_scores = attn_scores + rel_pos_bias.unsqueeze(0)

if mask is not None:

attn_scores = attn_scores.masked_fill(mask == 0, float('-inf'))

attn_weights = F.softmax(attn_scores, dim=-1)

attn_weights = self.dropout(attn_weights)

attn_output = torch.matmul(attn_weights, V)

attn_output = attn_output.transpose(1, 2).contiguous().view(batch_size, seq_len, self.hidden_dim)

output = self.out_proj(attn_output)

output = self.layer_norm(output + x)

return output, attn_weights

class DynamicGraphConstructor(nn.Module):

"""Dynamic graph constructor based on patient similarity."""

def __init__(self, hidden_dim, sigma=2.0, tau=0.3):

super(DynamicGraphConstructor, self).__init__()

self.sigma = sigma

self.tau = tau

self.similarity_net = nn.Sequential(

nn.Linear(hidden_dim * 2, hidden_dim),

nn.ReLU(),

nn.Linear(hidden_dim, 1),

nn.Sigmoid()

)

def forward(self, node_features):

# node_features: [num_nodes, hidden_dim]

num_nodes = node_features.size(0)

# Compute pairwise similarities

feat_i = node_features.unsqueeze(1).expand(-1, num_nodes, -1)

feat_j = node_features.unsqueeze(0).expand(num_nodes, -1, -1)

pair_features = torch.cat([feat_i, feat_j], dim=-1)

clinical_sim = self.similarity_net(pair_features).squeeze(-1)

# Gaussian kernel on feature distance

dist = torch.cdist(node_features, node_features, p=2)

gaussian_sim = torch.exp(-dist ** 2 / (2 * self.sigma ** 2))

# Combined similarity with thresholding

combined_sim = gaussian_sim * (clinical_sim > self.tau).float()

# Convert to edge index and weights

edge_index = (combined_sim > 0).nonzero(as_tuple=False).t()

edge_weight = combined_sim[edge_index[0], edge_index[1]]

return edge_index, edge_weight

class MSTGCANet(nn.Module):

"""Multimodal Spatiotemporal Graph Convolutional Attention Network."""

def __init__(

self,

vital_dim=12,

lab_dim=48,

imaging_dim=512,

text_dim=768,

rehab_dim=24,

hidden_dim=256,

num_classes=4,

num_graph_layers=3,

num_temporal_layers=2,

num_heads=8,

max_seq_len=12,

dropout=0.3

):

super(MSTGCANet, self).__init__()

# Modality-specific encoders

self.vital_encoder = ModalityEncoder(vital_dim, hidden_dim, 'numeric')

self.lab_encoder = ModalityEncoder(lab_dim, hidden_dim, 'numeric')

self.imaging_encoder = ModalityEncoder(imaging_dim, hidden_dim, 'imaging')

self.text_encoder = ModalityEncoder(text_dim, hidden_dim, 'text')

self.rehab_encoder = ModalityEncoder(rehab_dim, hidden_dim, 'numeric')

# Multimodal fusion

self.modality_fusion = AdaptiveModalityFusion(hidden_dim, num_modalities=5, num_heads=num_heads)

# Dynamic graph constructor

self.graph_constructor = DynamicGraphConstructor(hidden_dim)

# Spatial graph encoder

self.spatial_encoder = SpatialGraphEncoder(hidden_dim, hidden_dim, num_graph_layers)

# Temporal attention encoder

self.temporal_layers = nn.ModuleList([

TemporalAttentionEncoder(hidden_dim, num_heads, max_seq_len)

for _ in range(num_temporal_layers)

])

# Risk classification head

self.classifier = nn.Sequential(

nn.Linear(hidden_dim, hidden_dim),

nn.LayerNorm(hidden_dim),

nn.ReLU(),

nn.Dropout(dropout),

nn.Linear(hidden_dim, hidden_dim // 2),

nn.ReLU(),

nn.Dropout(dropout),

nn.Linear(hidden_dim // 2, num_classes)

)

# Intervention generation head

self.intervention_decoder = InterventionDecoder(hidden_dim, num_classes)

self.dropout = nn.Dropout(dropout)

def encode_modalities(self, vital, lab, imaging, text, rehab):

vital_feat = self.vital_encoder(vital)

lab_feat = self.lab_encoder(lab)

imaging_feat = self.imaging_encoder(imaging)

text_feat = self.text_encoder(text)

rehab_feat = self.rehab_encoder(rehab)

return [vital_feat, lab_feat, imaging_feat, text_feat, rehab_feat]

def forward(self, vital, lab, imaging, text, rehab, return_attention=False):

batch_size, seq_len = vital.size(0), vital.size(1)

# Process each time step

all_fused = []

modality_weights_all = []

for t in range(seq_len):

modality_features = self.encode_modalities(

vital[:, t], lab[:, t], imaging[:, t], text[:, t], rehab[:, t]

)

fused, weights = self.modality_fusion(modality_features)

all_fused.append(fused)

modality_weights_all.append(weights)

# Stack temporal features

temporal_features = torch.stack(all_fused, dim=1) # [batch, seq_len, hidden]

# Temporal attention encoding

temporal_attention_weights = []

for temporal_layer in self.temporal_layers:

temporal_features, attn_weights = temporal_layer(temporal_features)

temporal_attention_weights.append(attn_weights)

# Use last time step for graph construction and classification

final_features = temporal_features[:, -1] # [batch, hidden]

# Dynamic graph construction

edge_index, edge_weight = self.graph_constructor(final_features)

# Spatial graph encoding

graph_features = self.spatial_encoder(final_features, edge_index, edge_weight)

# Classification

logits = self.classifier(graph_features)

risk_probs = F.softmax(logits, dim=-1)

# Intervention generation

interventions = self.intervention_decoder(graph_features, risk_probs)

if return_attention:

return logits, interventions, {

'modality_weights': torch.stack(modality_weights_all, dim=1),

'temporal_attention': temporal_attention_weights,

'edge_index': edge_index,

'edge_weight': edge_weight

}

return logits, interventions

class InterventionDecoder(nn.Module):

"""Constrained intervention sequence decoder."""

def __init__(self, hidden_dim, num_risk_classes, vocab_size=108, max_interventions=8):

super(InterventionDecoder, self).__init__()

self.vocab_size = vocab_size

self.max_interventions = max_interventions

self.risk_embedding = nn.Linear(num_risk_classes, hidden_dim)

self.intervention_embedding = nn.Embedding(vocab_size + 2, hidden_dim) # +2 for SOS, EOS

self.decoder_lstm = nn.LSTM(hidden_dim * 2, hidden_dim, num_layers=2, batch_first=True)

self.output_proj = nn.Linear(hidden_dim, vocab_size + 2)

# Constraint matrix (to be loaded)

self.register_buffer('constraint_matrix', torch.ones(vocab_size, vocab_size))

def forward(self, patient_features, risk_probs, target_interventions=None):

batch_size = patient_features.size(0)

risk_feat = self.risk_embedding(risk_probs)

context = torch.cat([patient_features, risk_feat], dim=-1)

if self.training and target_interventions is not None:

# Teacher forcing

embedded = self.intervention_embedding(target_interventions)

decoder_input = torch.cat([embedded, context.unsqueeze(1).expand(-1, embedded.size(1), -1)], dim=-1)

output, _ = self.decoder_lstm(decoder_input)

logits = self.output_proj(output)

return logits

else:

# Autoregressive generation

generated = []

hidden = None

current_token = torch.zeros(batch_size, dtype=torch.long, device=patient_features.device) # SOS

for _ in range(self.max_interventions):

embedded = self.intervention_embedding(current_token).unsqueeze(1)

decoder_input = torch.cat([embedded, context.unsqueeze(1)], dim=-1)

output, hidden = self.decoder_lstm(decoder_input, hidden)

logits = self.output_proj(output.squeeze(1))

# Apply constraints

if len(generated) > 0:

for prev_token in generated:

constraint_mask = self.constraint_matrix[prev_token]

logits = logits.masked_fill(constraint_mask == 0, float('-inf'))

current_token = logits.argmax(dim=-1)

generated.append(current_token)

if (current_token == self.vocab_size + 1).all(): # EOS

break

return torch.stack(generated, dim=1)

class MSTGCANetTrainer:

"""Training utilities for MSTGCA-Net."""

def __init__(self, model, device, learning_rate=1e-4, weight_decay=1e-5):

self.model = model.to(device)

self.device = device

self.optimizer = torch.optim.AdamW(

model.parameters(),

lr=learning_rate,

weight_decay=weight_decay

)

self.scheduler = torch.optim.lr_scheduler.CosineAnnealingLR(

self.optimizer, T_max=200, eta_min=1e-6

)

self.ce_loss = nn.CrossEntropyLoss()

self.nll_loss = nn.NLLLoss(ignore_index=-1)

# Loss weights

self.lambda_1 = 1.0 # Classification

self.lambda_2 = 0.5 # Intervention generation

self.lambda_3 = 0.01 # Regularization

def train_step(self, batch):

self.model.train()

self.optimizer.zero_grad()

vital = batch['vital'].to(self.device)

lab = batch['lab'].to(self.device)

imaging = batch['imaging'].to(self.device)

text = batch['text'].to(self.device)

rehab = batch['rehab'].to(self.device)

risk_labels = batch['risk_label'].to(self.device)

intervention_labels = batch['interventions'].to(self.device)

logits, intervention_logits = self.model(vital, lab, imaging, text, rehab)

# Classification loss

loss_ce = self.ce_loss(logits, risk_labels)

# Intervention generation loss

loss_nll = self.nll_loss(

intervention_logits.view(-1, intervention_logits.size(-1)),

intervention_labels.view(-1)

)

# Regularization

loss_reg = sum(p.pow(2).sum() for p in self.model.parameters())

total_loss = self.lambda_1 * loss_ce + self.lambda_2 * loss_nll + self.lambda_3 * loss_reg

total_loss.backward()

torch.nn.utils.clip_grad_norm_(self.model.parameters(), max_norm=1.0)

self.optimizer.step()

return {

'total_loss': total_loss.item(),

'ce_loss': loss_ce.item(),

'nll_loss': loss_nll.item()

}

@torch.no_grad()

def evaluate(self, dataloader):

self.model.eval()

all_preds = []

all_labels = []

for batch in dataloader:

vital = batch['vital'].to(self.device)

lab = batch['lab'].to(self.device)

imaging = batch['imaging'].to(self.device)

text = batch['text'].to(self.device)

rehab = batch['rehab'].to(self.device)

logits, _ = self.model(vital, lab, imaging, text, rehab)

preds = logits.argmax(dim=-1)

all_preds.extend(preds.cpu().numpy())

all_labels.extend(batch['risk_label'].numpy())

return np.array(all_preds), np.array(all_labels)

@torch.no_grad()

def predict_with_uncertainty(self, batch, num_samples=50):

"""Monte Carlo dropout for uncertainty estimation."""

self.model.train() # Enable dropout

vital = batch['vital'].to(self.device)

lab = batch['lab'].to(self.device)

imaging = batch['imaging'].to(self.device)

text = batch['text'].to(self.device)

rehab = batch['rehab'].to(self.device)

predictions = []

for _ in range(num_samples):

logits, _ = self.model(vital, lab, imaging, text, rehab)

probs = F.softmax(logits, dim=-1)

predictions.append(probs.cpu())

predictions = torch.stack(predictions, dim=0)

mean_pred = predictions.mean(dim=0)

std_pred = predictions.std(dim=0)

# Predictive entropy

entropy = -(mean_pred * torch.log(mean_pred + 1e-8)).sum(dim=-1)

return {

'mean_prediction': mean_pred,

'std_prediction': std_pred,

'entropy': entropy

}

**Supplementary Code S2. Data Preprocessing Pipeline**

import numpy as np

import pandas as pd

from sklearn.preprocessing import StandardScaler, MinMaxScaler

from sklearn.impute import SimpleImputer

from transformers import AutoTokenizer, AutoModel

import torch

class RareDiseaseDataPreprocessor:

"""Preprocessing pipeline for rare disease rehabilitation data."""

def __init__(self, disease_categories_path=None):

self.vital_scaler = StandardScaler()

self.lab_scaler = MinMaxScaler()

# Disease-specific imputers

self.disease_imputers = {}

# Clinical BERT for text encoding

self.tokenizer = AutoTokenizer.from_pretrained('emilyalsentzer/Bio_ClinicalBERT')

self.bert_model = AutoModel.from_pretrained('emilyalsentzer/Bio_ClinicalBERT')

if disease_categories_path:

self.disease_categories = pd.read_csv(disease_categories_path)

def fit(self, data_dict, disease_labels):

"""Fit preprocessing transformations on training data."""

# Fit scalers

vital_flat = data_dict['vital'].reshape(-1, data_dict['vital'].shape[-1])

self.vital_scaler.fit(vital_flat)

lab_flat = data_dict['lab'].reshape(-1, data_dict['lab'].shape[-1])

self.lab_scaler.fit(lab_flat)

# Fit disease-specific imputers

unique_diseases = np.unique(disease_labels)

for disease in unique_diseases:

mask = disease_labels == disease

disease_data = lab_flat[mask.flatten()]

imputer = SimpleImputer(strategy='median')

imputer.fit(disease_data)

self.disease_imputers[disease] = imputer

return self

def transform(self, data_dict, disease_labels=None):

"""Apply preprocessing transformations."""

processed = {}

# Vital signs: Z-score normalization

vital = data_dict['vital'].copy()

original_shape = vital.shape

vital_flat = vital.reshape(-1, vital.shape[-1])

vital_flat = self.vital_scaler.transform(vital_flat)

processed['vital'] = vital_flat.reshape(original_shape)

# Laboratory: disease-stratified imputation + MinMax scaling

lab = data_dict['lab'].copy()

lab_shape = lab.shape

lab_flat = lab.reshape(-1, lab.shape[-1])

if disease_labels is not None:

for disease in np.unique(disease_labels):

mask = disease_labels == disease

if disease in self.disease_imputers:

lab_flat[mask.flatten()] = self.disease_imputers[disease].transform(

lab_flat[mask.flatten()]

)

lab_flat = self.lab_scaler.transform(lab_flat)

processed['lab'] = lab_flat.reshape(lab_shape)

# Imaging: assumed already CNN-encoded

processed['imaging'] = data_dict['imaging']

# Text: BERT encoding

if 'text' in data_dict:

processed['text'] = self.encode_clinical_notes(data_dict['text'])

# Rehabilitation scales: ordinal encoding (preserve order)

processed['rehab'] = data_dict['rehab'].copy()

return processed

def encode_clinical_notes(self, notes):

"""Encode clinical notes using ClinicalBERT."""

embeddings = []

self.bert_model.eval()

with torch.no_grad():

for note in notes:

if isinstance(note, float) and np.isnan(note):

# Missing note: use zero embedding with special marker

embeddings.append(np.zeros(768))

continue

inputs = self.tokenizer(

note,

padding='max_length',

truncation=True,

max_length=512,

return_tensors='pt'

)

outputs = self.bert_model(**inputs)

cls_embedding = outputs.last_hidden_state[:, 0, :].squeeze().numpy()

embeddings.append(cls_embedding)

return np.array(embeddings)

def handle_missing_values(self, data, strategy='disease_stratified'):

"""Handle missing values with multiple strategies."""

if strategy == 'disease_stratified':

# Use disease-specific medians

return self._disease_stratified_impute(data)

elif strategy == 'locf':

# Last observation carried forward

return self._locf_impute(data)

elif strategy == 'mice':

# Multiple imputation by chained equations

return self._mice_impute(data)

else:

raise ValueError(f"Unknown imputation strategy: {strategy}")

def _locf_impute(self, data):

"""Last observation carried forward imputation."""

df = pd.DataFrame(data)

return df.fillna(method='ffill').fillna(method='bfill').values

def detect_outliers(self, data, contamination=0.05):

"""Detect outliers using Isolation Forest."""

from sklearn.ensemble import IsolationForest

iso_forest = IsolationForest(contamination=contamination, random_state=42)

outlier_labels = iso_forest.fit_predict(data)

return outlier_labels == -1 # True for outliers

class PatientGraphConstructor:

"""Construct patient similarity graphs."""

def __init__(self, sigma=2.0, tau=0.3):

self.sigma = sigma

self.tau = tau

def compute_clinical_similarity(self, features, domain_weights=None):

"""Compute composite clinical similarity between patients."""

if domain_weights is None:

domain_weights = {

'diagnosis': 0.3,

'laboratory': 0.25,

'medication': 0.25,

'functional': 0.2

}

# Cosine similarity for each domain

similarities = {}

for domain, weight in domain_weights.items():

if domain in features:

domain_feat = features[domain]

norm_feat = domain_feat / (np.linalg.norm(domain_feat, axis=1, keepdims=True) + 1e-8)

sim = np.dot(norm_feat, norm_feat.T)

similarities[domain] = weight * sim

# Weighted combination

composite_sim = sum(similarities.values())

return composite_sim

def build_graph(self, patient_embeddings, clinical_similarity):

"""Build patient relationship graph."""

num_patients = patient_embeddings.shape[0]

# Gaussian kernel on embedding distance

dist = np.linalg.norm(

patient_embeddings[:, np.newaxis] - patient_embeddings[np.newaxis, :],

axis=-1

)

gaussian_sim = np.exp(-dist ** 2 / (2 * self.sigma ** 2))

# Apply clinical similarity threshold

mask = clinical_similarity > self.tau

# Final edge weights

edge_weights = gaussian_sim * mask

# Convert to edge list

edge_index = np.array(np.nonzero(edge_weights))

edge_weight = edge_weights[edge_index[0], edge_index[1]]

return {

'edge_index': edge_index,

'edge_weight': edge_weight,

'adjacency_matrix': edge_weights,

'graph_density': np.sum(mask) / (num_patients ** 2)

}

**Supplementary Code S3. Evaluation Metrics Implementation**

import numpy as np

from sklearn.metrics import (

accuracy_score, precision_score, recall_score, f1_score,

roc_auc_score, confusion_matrix, cohen_kappa_score,

classification_report

)

from scipy import stats

import warnings

class RiskStratificationEvaluator:

"""Comprehensive evaluation metrics for risk stratification."""

def __init__(self, num_classes=4, class_names=None):

self.num_classes = num_classes

self.class_names = class_names or ['High', 'Moderate-High', 'Moderate-Low', 'Low']

def compute_all_metrics(self, y_true, y_pred, y_prob=None):

"""Compute all classification metrics."""

results = {

'accuracy': accuracy_score(y_true, y_pred),

'precision_macro': precision_score(y_true, y_pred, average='macro', zero_division=0),

'recall_macro': recall_score(y_true, y_pred, average='macro', zero_division=0),

'f1_macro': f1_score(y_true, y_pred, average='macro', zero_division=0),

'f1_weighted': f1_score(y_true, y_pred, average='weighted', zero_division=0),

'kappa': cohen_kappa_score(y_true, y_pred),

'confusion_matrix': confusion_matrix(y_true, y_pred)

}

# Per-class metrics

for i, name in enumerate(self.class_names):

binary_true = (y_true == i).astype(int)

binary_pred = (y_pred == i).astype(int)

results[f'{name}_precision'] = precision_score(binary_true, binary_pred, zero_division=0)

results[f'{name}_recall'] = recall_score(binary_true, binary_pred, zero_division=0)

results[f'{name}_f1'] = f1_score(binary_true, binary_pred, zero_division=0)

# AUC if probabilities available

if y_prob is not None:

try:

results['auc_macro'] = roc_auc_score(

y_true, y_prob, multi_class='ovr', average='macro'

)

results['auc_weighted'] = roc_auc_score(

y_true, y_prob, multi_class='ovr', average='weighted'

)

# Per-class AUC

for i, name in enumerate(self.class_names):

binary_true = (y_true == i).astype(int)

results[f'{name}_auc'] = roc_auc_score(binary_true, y_prob[:, i])

except ValueError as e:

warnings.warn(f"Could not compute AUC: {e}")

return results

def bootstrap_confidence_interval(self, y_true, y_pred, y_prob=None,

n_iterations=1000, ci=0.95):

"""Compute bootstrap confidence intervals for metrics."""

n_samples = len(y_true)

metrics_list = []

for _ in range(n_iterations):

indices = np.random.choice(n_samples, n_samples, replace=True)

y_true_boot = y_true[indices]

y_pred_boot = y_pred[indices]

y_prob_boot = y_prob[indices] if y_prob is not None else None

metrics = self.compute_all_metrics(y_true_boot, y_pred_boot, y_prob_boot)

metrics_list.append(metrics)

# Compute confidence intervals

ci_results = {}

alpha = (1 - ci) / 2

for key in ['accuracy', 'f1_macro', 'auc_macro', 'precision_macro', 'recall_macro']:

if key in metrics_list[0]:

values = [m[key] for m in metrics_list]

ci_results[key] = {

'mean': np.mean(values),

'std': np.std(values),

'ci_lower': np.percentile(values, alpha * 100),

'ci_upper': np.percentile(values, (1 - alpha) * 100)

}

return ci_results

def statistical_comparison(self, metrics_a, metrics_b, test='wilcoxon'):

"""Statistical comparison between two models."""

if test == 'wilcoxon':

stat, p_value = stats.wilcoxon(metrics_a, metrics_b)

elif test == 'paired_t':

stat, p_value = stats.ttest_rel(metrics_a, metrics_b)

else:

raise ValueError(f"Unknown test: {test}")

return {

'statistic': stat,

'p_value': p_value,

'significant_0.05': p_value < 0.05,

'significant_bonferroni': p_value < 0.05 / 28 # Bonferroni correction

}

class InterventionEvaluator:

"""Evaluation metrics for intervention generation."""

def __init__(self, vocab_size=108):

self.vocab_size = vocab_size

def compute_bleu(self, references, hypotheses, max_n=4):

"""Compute BLEU scores."""

from nltk.translate.bleu_score import sentence_bleu, SmoothingFunction

smoother = SmoothingFunction().method1

bleu_scores = {f'bleu_{i}': [] for i in range(1, max_n + 1)}

for ref, hyp in zip(references, hypotheses):

for n in range(1, max_n + 1):

weights = tuple([1/n] * n + [0] * (4 - n))

score = sentence_bleu([ref], hyp, weights=weights, smoothing_function=smoother)

bleu_scores[f'bleu_{n}'].append(score)

return {k: np.mean(v) for k, v in bleu_scores.items()}

def compute_constraint_satisfaction(self, interventions, constraint_matrix):

"""Compute constraint satisfaction rate."""

total_pairs = 0

satisfied_pairs = 0

for intervention_seq in interventions:

for i, int_i in enumerate(intervention_seq):

for j, int_j in enumerate(intervention_seq):

if i < j and int_i < self.vocab_size and int_j < self.vocab_size:

total_pairs += 1

if constraint_matrix[int_i, int_j] == 1:

satisfied_pairs += 1

return satisfied_pairs / total_pairs if total_pairs > 0 else 1.0

def compute_clinical_validity(self, interventions, risk_categories, validity_rules):

"""Compute clinical validity rate based on expert-defined rules."""

valid_count = 0

total_count = len(interventions)

for intervention, risk in zip(interventions, risk_categories):

# Check if interventions are appropriate for risk level

valid = self._check_validity_rules(intervention, risk, validity_rules)

if valid:

valid_count += 1

return valid_count / total_count

def _check_validity_rules(self, intervention, risk_category, rules):

"""Check intervention against validity rules."""

# Required interventions for high risk

if risk_category == 0: # High risk

required = rules.get('high_risk_required', [])

if not any(req in intervention for req in required):

return False

# Contraindicated interventions

contraindicated = rules.get('contraindicated', {})

for int_id in intervention:

if int_id in contraindicated.get(risk_category, []):

return False

return True

def format_results_table(results_dict, format_type='latex'):

"""Format results dictionary as publication-ready table."""

if format_type == 'latex':

lines = [

"\\begin{table}[h]",

"\\centering",

"\\caption{Performance Comparison}",

"\\begin{tabular}{lcccc}",

"\\hline",

"Method & Accuracy & F1 Score & AUC & p-value \\\\",

"\\hline"

]

for method, metrics in results_dict.items():

line = f"{method} & {metrics['accuracy']:.3f} & {metrics['f1']:.3f} & {metrics['auc']:.3f} & {metrics.get('p_value', '-')} \\\\"

lines.append(line)

lines.extend([

"\\hline",

"\\end{tabular}",

"\\end{table}"

])

return '\n'.join(lines)

elif format_type == 'markdown':

lines = [

"| Method | Accuracy | F1 Score | AUC | p-value |",

"|--------|----------|----------|-----|---------|"

]

for method, metrics in results_dict.items():

line = f"| {method} | {metrics['accuracy']:.3f} | {metrics['f1']:.3f} | {metrics['auc']:.3f} | {metrics.get('p_value', '-')} |"

lines.append(line)

return '\n'.join(lines)

**Supplementary Table S16. Complete Constraint Matrix for Intervention Generation**

| **Category A** | **Category B** | **Constraint Type** | **Clinical Rationale** |
| --- | --- | --- | --- |
| Increase exercise | Bed rest | Incompatible | Activity level conflict |
| Add anticoagulant | High-intensity PT | Conditional | Bleeding risk |
| Increase opioid | Discharge home | Conditional | Safety monitoring required |
| Add sedative | Driving clearance | Incompatible | Impairment risk |
| NPO order | Oral medication | Incompatible | Administration conflict |
| ICU transfer | Discharge planning | Incompatible | Care level conflict |
| Cardiac rehab | Active infection | Conditional | Infection resolution required |
| Weight bearing | Fracture healing | Conditional | Healing status dependent |
| Immunosuppressant | Live vaccine | Incompatible | Contraindication |
| Increase fluids | Fluid restriction | Incompatible | Fluid management conflict |
| High protein diet | Renal restriction | Conditional | Kidney function dependent |
| PT frequency increase | Severe fatigue | Conditional | Tolerance assessment |
| Add stimulant | Sleep protocol | Conditional | Timing coordination |
| Increase activity | Oxygen dependency | Conditional | Oxygenation monitoring |
| Home discharge | 24h monitoring need | Incompatible | Monitoring requirement |

**Supplementary Table S17. Detailed Attention Weight Analysis by Modality and Time**

| **Time Point (Week)** | **Vital Signs** | **Laboratory** | **Imaging** | **Clinical Notes** | **Rehab Scales** |
| --- | --- | --- | --- | --- | --- |
| 1 (Acute) | 0.31 ± 0.08 | 0.28 ± 0.07 | 0.18 ± 0.06 | 0.12 ± 0.04 | 0.11 ± 0.04 |
| 2 | 0.28 ± 0.07 | 0.26 ± 0.06 | 0.19 ± 0.05 | 0.14 ± 0.05 | 0.13 ± 0.04 |
| 3 | 0.25 ± 0.06 | 0.24 ± 0.06 | 0.20 ± 0.05 | 0.16 ± 0.05 | 0.15 ± 0.05 |
| 4 | 0.22 ± 0.06 | 0.22 ± 0.05 | 0.21 ± 0.05 | 0.18 ± 0.05 | 0.17 ± 0.05 |
| 6 | 0.19 ± 0.05 | 0.20 ± 0.05 | 0.21 ± 0.05 | 0.20 ± 0.06 | 0.20 ± 0.05 |
| 8 | 0.16 ± 0.05 | 0.18 ± 0.05 | 0.20 ± 0.05 | 0.22 ± 0.06 | 0.24 ± 0.06 |
| 10 | 0.14 ± 0.04 | 0.16 ± 0.05 | 0.19 ± 0.05 | 0.24 ± 0.06 | 0.27 ± 0.07 |
| 12 (Discharge) | 0.12 ± 0.04 | 0.14 ± 0.04 | 0.18 ± 0.05 | 0.26 ± 0.07 | 0.30 ± 0.08 |

**Supplementary Table S18. Model Performance Under Different Missing Data Rates**

| **Missing Rate** | **Accuracy** | **F1 Score** | **AUC** | **Δ Performance** |
| --- | --- | --- | --- | --- |
| 0% (Complete) | 0.879 | 0.861 | 0.934 | — |
| 5% | 0.872 | 0.854 | 0.928 | -0.8% |
| 10% | 0.867 | 0.845 | 0.923 | -1.8% |
| 15% | 0.858 | 0.836 | 0.915 | -2.9% |
| 20% | 0.846 | 0.823 | 0.904 | -4.4% |
| 25% | 0.831 | 0.807 | 0.889 | -6.3% |
| 30% | 0.812 | 0.786 | 0.871 | -8.7% |
